# Novel Quinazolinones Active against Multidrug‐Resistant *Mycobacterium Tuberculosis:* Synthesis, Antimicrobial Evaluation, and in Silico Exploration of Penicillin‐Binding Protein 1A as a Potential Target

**DOI:** 10.1002/cmdc.202500147

**Published:** 2025-06-08

**Authors:** Marek Kerda, Daria Nawrot, Petr Šlechta, Miroslav Domanský, Asal Askari, Hanieh Kamangar, Ondřej Janďourek, Klára Konečná, Pavla Paterová, Ingrid Hlbočanová, Miloslav Macháček, Matteo Mori, Fiorella Meneghetti, Martin Doležal, Jan Zitko, Ghada Bouz

**Affiliations:** ^1^ Faculty of Pharmacy in Hradec Králové Charles University 500 05 Hradec Králové Czech Republic; ^2^ Department of Clinical Microbiology University Hospital Hradec Králové 500 05 Hradec Králové Czech Republic; ^3^ Department of Pharmaceutical Sciences Università degli Studi di Milano 20133 Milano Italy

**Keywords:** antimycobacterial, computational chemistries, drug designs, medicinal chemistries, multidrug‐resistant tuberculosis, penicillin‐binding proteins, quinazolinones

## Abstract

Quinazolinone derivatives have emerged as promising scaffolds in antimicrobial drug discovery. This work focuses on the design, synthesis, and evaluation of novel quinazolinone‐based compounds and predicts their potential to interact with mycobacterial penicillin‐binding proteins (PBPs). Relying on established structure–activity relationships of antibacterial quinazolinones, a total of 53 compounds belonging to three different structural types are synthesized and biologically evaluated for antimycobacterial, antibacterial, and antifungal activities. Biological evaluations reveal selective efficacy against *Mycobacterium tuberculosis* with minimum inhibitory concentrations (MICs) as low as 6.25 μg mL^−1^ for some derivatives, and this activity is preserved against drug‐resistant strains. Molecular docking studies suggest a potential allosteric binding site in mycobacterial PBP 1A (PonA1, UniProt ID: P71707), and subsequential molecular dynamics confirm stable binding with key stabilizing interaction between the carbonyl oxygen of the quinazolinone and either ARG399 or ASP474. These findings suggest quinazolinone derivatives as viable candidates for further development as non‐β‐lactam PBP inhibitors, addressing the urgent need for new antitubercular therapies.

## Graphical abstract

Quinazolinone‐based compounds emerge as potent antimycobacterial agents, showing low minimum inhibitory concentrations against drug‐resistant *Mycobacterium tuberculosis*. Molecular modeling uncovers a novel allosteric site in PonA1 targeted by these derivatives. Stable interactions with key residues suggest a non‐β‐lactam inhibition mechanism, positioning quinazolinones as powerful candidates in the fight against persistent tuberculosis.

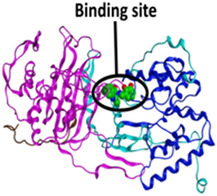

## 1 Introduction and Design Rationale

**Figure 1:**
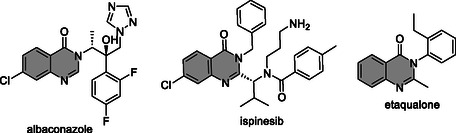
The chemical structures of clinically available quinazolinone‐containing drugs with different biological activities. The antifungal albaconazole inhibits lanosterol 14α demethylase; the anticancer agent ispinesib inhibits kinesin‐like protein 1; and the sedative etaqualone interacts with β‐subtype of the GABA_A_ receptor. The quinazolinone nucleus is shown in gray.

Quinazolin‐4(3*H*)‐one (referred to as quinazolinone), similar to its closely related cores quinoline and quinazoline, is an important pharmacophore and privileged structure known for its characteristic stability against oxidation, reduction, and hydrolysis.^[^ ^1^, ^2^, ^3^ ^]^ Quinazolinone‐containing compounds have gained importance in medicinal chemistry as they possess a wide spectrum of biological activities like antibacterial,^[^ ^4^ ^]^ antitubercular,^[^ ^5^, ^6^ ^]^ antimalarial,^[^ ^7^, ^8^ ^]^ anticonvulsant,^[^ ^9^ ^]^ antidepressant,^[^ ^10^ ^]^ and anticancer.^[^ ^11^, ^12^ ^]^ **Figure** **1** provides some examples of clinically available quinazolinone‐containing drugs with different biological activities.

**Figure 2:**
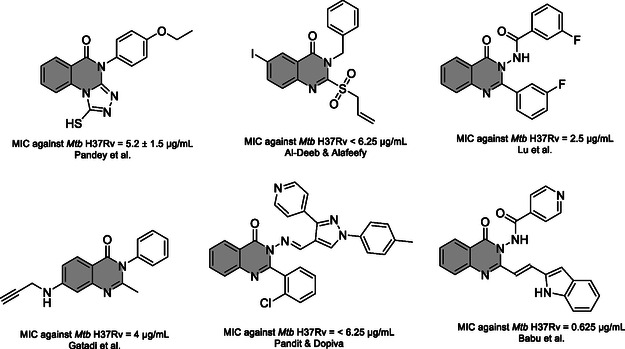
Reported quinazolinone‐containing compounds exerting antimycobacterial activity with their MICs.^[^ ^4^, ^5^, ^6^, ^18^, ^19^, ^20^ ^]^

In the context of antimicrobial research, quinazolinones are of particular interest. Several quinazolinone‐containing molecules have shown potent antibacterial activity against Gram‐positive and/or Gram‐negative bacteria.^[^ ^13^, ^14^, ^15^, ^16^ ^]^ Furthermore, albaconazole is an example of a clinically available antifungal quinazolinone. According to the World Health Organization, tuberculosis in 2023 has returned back as the leading cause of death from a single infectious agent.^[^ ^17^ ^]^ The suboptimal performance of available antituberculars creates an urgent need to develop novel agents, preferably with novel chemical structures and mechanisms of action. Several quinazolinone‐containing structures showed in vitro activity against *Mycobacterium tuberculosis* (*Mtb*).^[^ ^4^, ^5^, ^6^, ^18^, ^19^, ^20^ ^]^ Selected compounds with their minimum inhibitory concentrations (MIC) against *Mtb* H37Rv are displayed in **Figure** **2**.

**Figure 3:**
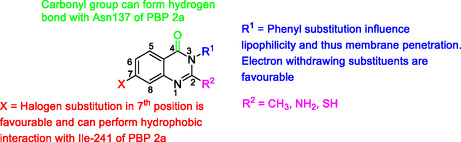
Quinazolinone nucleus with known favorable substitutions improving antimicrobial activity by targeting PBP.

Regarding their molecular targets, antibacterial quinazolinones were found to target DNA gyrase, dihydrofolate reductase, tyrosyl‐tRNA synthetase, and penicillin‐binding proteins (PBPs)—the focus of this article. Detailed information on the interactions of quinazolinone derivatives with end molecular targets can be found in the review by Samotrueva et al.^[^ ^21^ ^]^ PBPs are enzymes crucial for bacterial cell wall synthesis. PBPs are responsible for catalyzing the final stages of peptidoglycan biosynthesis, which is essential for maintaining bacterial cell wall integrity.^[^ ^22^ ^]^ Therefore, inhibiting PBPs leads to cell lysis and bacterial death. In general, certain structural modifications were found to enhance the observed antibacterial activity of quinazolinones targeting PBPs, leading to established structure‐activity‐relationships. Those include the substitution at position 2 of the quinazolinone nucleus, preferably with methyl, amino, or thiol groups; or position 3, preferably with a substituted aromatic ring; the presence of halogen atoms at position 6 or 7 (**Figure** **3**).^[^ ^4^, ^23^ ^]^

**Figure 4:**
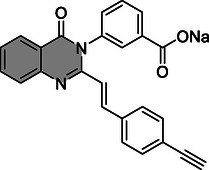
The chemical structure of the quinazolinone allosteric inhibitor of PBP 2a reported by Janardhanan et al. in Ref. [22].

In addition, it must be noted that quinazolinones as non‐β‐lactam inhibitors of PBPs can themselves potentiate the effects of other β‐lactam inhibitors of PBPs. In a murine in vivo model of methicillin‐resistant *Staphylococcus aureus* (MRSA) infection, a quinazolinone (refer to **Figure** **4** for chemical structure) was able to demonstrate synergism with piperacillin‐tazobactam by binding to PBP 2a allosteric site. PBP 2a is a major determinant of β‐lactam antibiotic resistance in MRSA as it has a low affinity for β‐lactam antibiotics.^[^ ^24^ ^]^ The reported binding induced an allosteric response that led to the active site opening *via* reorganisation of α2‐α3 loop, which, in return, allowed another molecule of piperacillin to bind, making PBP 2a more susceptible to inhibition by piperacillin.^[^ ^25^ ^]^

**Figure 5:**
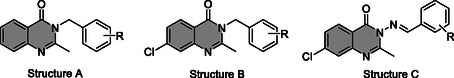
The three general structures of the title compounds. For R, refer to Table 1.

Based on our long‐term experience in developing potential antimycobacterials and the mentioned established structure–activity relationships of antimicrobial quinazolinones targeting PBPs, we report in this work the design, synthesis, and biological evaluation of a novel series of quinazolinones and their antimycobacterial activity evaluation against the pathogenic *Mtb* H37Rv and the surrogate model *Mtb* H37Ra. We designed three series of compounds bearing different substituents (**Figure** **5**) for general structures. Series A features a methylene linker (11 compounds), series B has an additional chlorine atom at C7 (13 compounds), series C bears an imine linker while preserving the chlorine atom at C7 (29 compounds). As complementary testing to explore the selectivity profile, the title compounds were screened against other mycobacterial strains, namely the fast‐growing *Mycolicibacterium smegmatis* (*M. smeg.*) and *Mycolicibacterium aurum* (*M. aurum*), along with the nontuberculous *Mycobacterium avium* (*M. avium*) and *Mycobacterium kansasii* (*M. kans.*). Complementary testing also included antibacterial evaluation against eight bacterial strains, four of which are Gram‐positive and four Gram‐negative, and antifungal evaluation against eight fungal strains.

## 2 Experimental Section

### 2.1 General Information

All reagents and solvents (unless stated otherwise) were purchased from Merck (Darmstadt, Germany) and used without further purification. Reaction progress and purity of products were monitored using Silica 60 F_254_ Thin‐Layer Chromatography (TLC) plates (Merck, Darmstadt, Germany). Flash chromatography of the final compounds was performed on a puriFlash XS420+ (Interchim, Montluçon, France) with original columns (spherical silica, 30 μm) provided by the same company. The mobile phase was ethyl acetate (EtOAc) in hexane (Hex), gradient elution 0–100%, and detection was performed by UV–vis detector at 254 and 280 nm. The NMR spectra were recorded on Jeol JNM‐ECZ600R at 600 MHz for ^1^H and 151 MHz for ^13^C at ambient temperature. The chemical shifts reported as δ values in ppm were indirectly referenced to tetramethylsilane (TMS) *via* the solvent signal (2.49 for ^1^H and 39.7 for ^13^C in DMSO‐*d*_6;_ 7.26 and 77.36 for CDCl_3_). IR spectra were recorded on a NICOLET 6700 FTIR spectrophotometer (Nicolet, Madison, WI, USA) using the attenuated total reflectance. Mass spectra in both positive and negative modes (Atmospheric Pressure Chemical Ionization‐APCI‐MS) were measured using the Expression compact mass spectrometer (Advion, Ithaca, NY, USA) with a single‐quad detector. Elemental analysis was done on a Vario MICRO cube element analyzer (Elementar Analysensysteme, Hanau, Germany) with values given as a percentage. Yields were given in percentage and refer to the amount of pure product after all purification steps. Log*P* values were calculated using ChemDraw v22.0. (PerkinElmer Informatics, Waltham, MA, USA).

### 2.2 Chemistry

#### 2.2.1 3‐Benzyl‐2‐methylquinazolin‐4(3*H*)‐ones (Series a, 1–11)

**Scheme 1:**
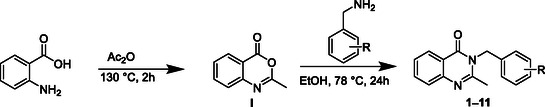
Synthesis of compounds from Series A (**1**–**11**).

Starting material anthranilic acid 10 g (73 mmol) was dissolved in 50 mL of Ac_2_O (excess) and heated to 130 °C for 2 h with stirring. The solvent was then evaporated under reduced pressure and the crude product was recrystallized in Hex/EtOAc 98:2, resulting in intermediate (**I**). Final products were prepared by reacting 1 mmol of intermediate (**I**) dissolved in EtOH and 1.2 mmol of respective benzylamine. The mixture was heated to 78 °C and refluxed for 24 h with stirring. After completion of the reaction (monitored by TLC), the solvent was evaporated under reduced pressure. The crude product was purified using flash chromatography, **Scheme** **1**.

#### 2.2.2 3‐Benzyl‐7‐chloro‐2‐methylquinazolin‐4(3*H*)‐ones (Series B, 12–24)

**Scheme 2:**
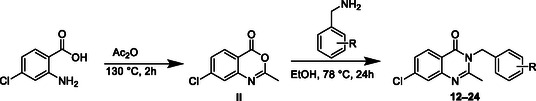
Synthesis of compounds from series B (**12**–**24**).

We followed the same procedure as in Section 2.2.1., except that 4‐chloroanthranilic acid was used as a starting material, leading to intermediate (**II**). The final products were also purified and analyzed in a similar fashion **Scheme** **2**.

#### 2.2.3 (E)‐3‐(benzylideneamino)‐7‐chloro‐2‐methylquinazolin‐4(3*H*)‐ones (Series C, 25–53)

**Scheme 3:**
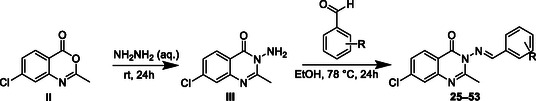
Synthesis of compounds from series C (**25**–**53**).

5 g of intermediate 2 (15.5 mmol) dissolved in 10 mL of 35% (m m^−1^) hydrazine in water (excess). The mixture was stirred for 24 h at room temperature, forming a white precipitate. The precipitate was filtered off and washed several times with water, resulting in pure intermediate (**III**), which was dried and used directly in the following step without further purification. 1 mmol of intermediate (**III**) was dissolved in EtOH and 1.1 equivalent of respective benzaldehyde was added. The reaction was heated to 78 °C and refluxed for 24 h with stirring. After completion of the reaction (monitored by TLC), the mixture was cooled down, and the formed precipitate was collected by filtration and washed with EtOH, resulting in the final products, as shown in **Scheme** **3**.

### 2.3 Analytical Data

The prepared final compounds were characterized by ^1^H and ^13^C NMR, IR spectroscopy, elemental analysis, melting point, and mass spectrometry. The acquired data were fully consistent with the proposed structures. In series C, the *E*‐configuration of the C=N double bond was confirmed by X‐ray crystallography of compound **47**. Complete characterization and analytical data are located in supplementary materials.

### 2.4 Biological Evaluations

Refer to the supplementary materials for full methodology.

### 2.5 In Silico Simulations

#### 2.5.1 Software

The molecular docking was performed in Molecular Operating Environment (MOE) 2022.02 (Chemical Computing Group, Montreal, QC, Canada) under Amber10:EHT forcefield if not otherwise stated. Molecular dynamics simulation was run with the same forcefield parameters using NAMD (University of Illinois at Urbana‐Champaign, IL, USA). Minimization and heating stages were simulated using NAMD 2.10, whereas the production phase was calculated using NAMD 3 alpha 9 utilizing CUDA GPU acceleration. Trajectory analysis was performed using Visual Molecular Dynamics (VMD) version 1.9.4a53, University of Illinois at Urbana‐Champaign, IL, USA).

#### 2.5.2 Molecular Docking

The 3D coordinates of PBP 1A (PonA1, UniProt ID: P71707) were downloaded as a pdb file from AlphaFold. Disordered parts of the *N*‐terminus (residues 1 to 152) and the *C*‐terminus (765 to 820) were removed. The system was prepared using MOE QuickPrep functionality with default settings, which included corrections of structural errors, the addition of hydrogens, calculation of partial charges, 3D optimization of protonation/tautomeric states and H‐bond network (Protonate3D), and a restrained minimization (to Root Mean Square (RMS) gradient of 0.01 kcal mol^−1^ Å^−1^). A potential binding site was created using Site Finder built‐in functionality in MOE, which calculates potential sites based on geometric properties and the interaction potential. Ligands for docking were drawn manually and converted to 3D. The predicted dominant protomer at pH 7.4 was minimized until RMS gradient 0.01 kcal mol^−1^ Å^−1^. Parameters of the MOE docking protocol: Docking stage–Placement: Triangle Matcher; score: London dG; retain 500 poses: Refinement stage–Rigid receptor; score: GBVI/WSA dG; retain 10 poses; Ligand conformation–Rotate bonds.

#### 2.5.3 Molecular Dynamics

Starting systems for the MD simulations were prepared in MOE, applying the parameters from Amber10:EHT forcefield. The system was solvated using TIP3P waters in a 10 Å margin periodic boundaries box, neutralized, and buffered using NaCl (c = 0.15 M). Nonbonded Van der Waals interactions were truncated (switching distance 10, cut‐off distance 12). Long‐range electrostatics were treated using the Particle Mesh Ewald (PME). Bonds to hydrogen atoms were constrained using the ShakeH algorithm (with a default convergence criterion of 1.0 × 10^−8^). The temperature was controlled by Langevin dynamics, and the pressure was treated using the Nosé–Hoover–Langevin piston pressure control. The time step was 2 fs, and the coordinates were recorded each 10 ps.

MD Protocol (time in ps, temperature *T* in Kelvins, pressure *P* in bar, *r* is a restraint to heavy atoms as defined in MOE, see below *).

Stage 1: Restrained minimization {ps = 10 *T* = 0 *r* = 0.5};

Stage 2: Unrestrained minimization {ps = 10 *T* = 0};

Stage 3: Heating with gradually released restraints {ps = 180 *T* = (10 300) *r* = (0.5,10)};

Stage 4: NVT equilibration {ps = 200 *T* = 300};

Stage 5: Fixed term for isothermal–isobaric ensemble (NPT) equilibration {ps = 10 600 *T* = 300 *P* = 1};

Stage 6: NPT production {ps = 100 000 *T* = 300 *P* = 1};

 * Restraint: Heavy atom tether restraint in Å. A value of 0 means that heavy atoms are fixed, while, for example, 1 means that a restraint force constant that produces a 1 Å radial standard deviation from the reference position will be applied.

The Root Mean Square Deviation (RMSD) analysis was done using the VMD plugin. Ligand–protein interactions were analyzed using protein–ligand interaction fingerprints (PLIF) function built in MOE. Hydrogen bond donor (HBD), hydrogen bond acceptor (HBA), and arene attraction between ligand and sidechains or backbone were generated. Only strong interactions defined as ≥1.5 kcal/mol for hydrogen bonds and ≥3.5 kcal/mol for ionic bonds were considered.

## 3 Results and Discussion

### 3.1 Antimycobacterial Activity

All compounds were screened for whole‐cell antimycobacterial activity using the Microplate Alamar Blue assay. Tested strains included *Mtb* H37Rv, avirulent *Mtb* H37Ra, fast‐growing model organism *M. smeg.* and *M. aurum,* nontuberculous *M. avium* and *M. kansasii.* Results are present in **Table** **1** as MIC in μg/mL. Activity cut‐off values were set to ≤25 μg mL^−1^ for *Mtb* H37Rv and *M. kans.* and ≤31.25 μg mL^−1^ for the remaining strains.

**Table 1:** Final compounds with calculated lipophilicity (log*P*) values and antimycobacterial activity expressed as MICs.

Cmpd.	R	log*P*	Antimycobacterial Activity MIC in μg mL^−1^					
			*Mtb* H37Rv	*Mtb* H37Ra	*M. kans.*	*M. avium*	*M. smeg.*	*M. aurum*
**Structure A**								
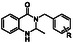								

1	2,4‐diOCH_3_	2.69	>100	≥500	>100	≥500	≥500	≥500
2	3‐OCH_3_	2.82	100	62.5	100	62.5	250	125
3	4‐OCH_3_	2.82	>100	≥250	>100	≥250	≥250	≥250
4	3‐F	3.1	100	250	50	125	250	500
5	2‐CH_3_	3.43	>100	≥500	>100	≥500	≥500	≥500
6	3‐Cl	3.5	50	**31.25**	**25**	**31.25**	62.5	62.5
7	4‐CF_3_	3.86	>100	62.5	>100	125	250	125
8	see **Figure** **6**	3.94	>100	≥250	50	62.5	≥500	62.5
9	3‐F, 5‐CF_3_	4.02	>100	125	>100	62.5	≥500	62.5
10	2,4‐diCl	4.06	>100	≥250	>100	≥250	≥250	≥250
11	3,4‐diCl	4.06	>100	≥250	>100	≥250	≥250	≥250
**Structure B**								
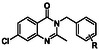								

12	3‐COOH	3.06	>100	≥62.5	>100	≥62.5	≥62.5	≥62.5
13	3‐OH	3.11	50	≥62.5	>100	62.5	≥62.5	≥62.5
14	4‐OH	3.11	>100	62.5	>100	62.5	62.5	62.5
15	2,4‐diOCH_3_	3.25	>100	≥125	>100	≥125	≥125	≥125
16	3‐OCH_3_	3.38	>100	62.5	>100	≥500	≥125	≥125
17	4‐OCH_3_	3.38	**12.5**	**7.81**	>100	≥500	**31.25**	**15.625**
18	2‐F	3.66	>100	≥500	>100	≥500	≥500	≥500
19	4‐F	3.66	**25**	**7.81**	>100	**15.625**	**31.25**	**31.25**
20	2‐CH_3_	3.99	>100	≥250	>100	≥250	≥250	≥250
21	3‐Cl	4.09	**25**	**15.625**	>100	≥250	≥250	≥250
22	4‐CF_3_	4.42	>100	**31.25**	**25**	≥500	≥500	≥500
23	See Figure 6	4.5	>100	≥125	>100	≥125	≥125	≥125
24	3,4‐diCl	4.62	50	**15.625**	**25**	≥125	≥125	≥125
**Structure C**								
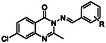								

25	3‐NO_2_ 4‐OH	n/a	**12.5**	**31.25**	>100	62.5	**31.25**	≥62.5
26	2‐NO_2_	n/a	>25	≥62.5	>25	≥62.5	≥62.5	≥62.5
27	3‐NO_2_	n/a	>50	≥62.5	>50	≥62.5	≥62.5	≥62.5
28	4‐NO_2_	n/a	>25	≥31.25	>25	≥31.25	≥31.25	≥31.25
29	see Figure 6	2.62	>25	≥31.25	>25	≥31.25	≥31.25	≥31.25
30	see Figure 6	2.62	>25	≥31.25	>25	≥31.25	≥31.25	≥31.25
31	see Figure 6	2.62	>25	≥31.25	>25	≥31.25	≥31.25	≥31.25
32	2,3‐diOH	3.18	**25**	**15.625**	**25**	≥62.5	≥62.5	≥62.5
33	2,4‐diOH	3.18	>100	≥62.5	>100	≥62.5	≥62.5	≥62.5
34	4‐OH 3‐OCH_3_	3.44	>100	≥62.5	>100	≥62.5	≥62.5	≥62.5
35	2‐OH	3.57	>50	≥31.25	>50	≥31.25	≥31.25	≥31.25
36	3‐OH	3.57	>100	≥62.5	>100	≥62.5	≥62.5	≥62.5
37	4‐OH	3.57	>50	≥62.5	>50	≥62.5	≥62.5	≥62.5
38	2‐OCH_3_	3.83	>25	≥62.5	>25	≥62.5	≥62.5	≥62.5
39	3‐OCH_3_	3.83	>50	≥62.5	>50	≥62.5	≥62.5	≥62.5
40	4‐OCH_3_	3.83	>50	≥15.625	>50	≥15.625	≥15.625	≥15.625
41	see Figure 6	3.84	>25	≥31.25	>25	≥31.25	≥31.25	≥31.25
42	2‐F	4.12	>25	≥31.25	>25	≥31.25	≥31.25	≥31.25
43	3‐F	4.12	>25	≥62.5	>25	≥62.5	≥62.5	≥62.5
44	4‐F	4.12	>25	≥31.25	>25	≥31.25	≥31.25	≥31.25
45	4‐N(CH_3_)_2_	4.24	>25	≥15.625	>25	≥15.625	≥15.625	≥15.625
46	2‐CH_3_	4.44	>25	≥62.5	>25	≥62.5	≥62.5	≥62.5
47	3‐CH_3_	4.44	>25	≥31.25	>25	≥31.25	≥31.25	≥31.25
48	4‐CH_3_	4.44	>25	≥31.25	>25	≥31.25	≥31.25	≥31.25
49	2‐Cl	4.52	>25	≥31.25	>25	≥31.25	≥31.25	≥31.25
50	3‐Cl	4.52	>50	≥62.5	>50	≥62.5	≥62.5	≥62.5
51	4‐Cl	4.52	>25	≥15.625	>25	≥15.625	≥15.625	≥15.625
52	2‐Br	4.79	>25	≥31.25	>25	≥31.25	≥31.25	≥31.25
53	3‐Br	4.79	>25	≥31.25	>25	≥31.25	≥31.25	≥31.25
INH			0.2	0.25	25	1000	15.625	3.91
RIF			n/a	0.0015625	0.025	0.125	12.5	0.39
CIP			n/a	0.25	0.25	1.56	0.125	0.015625

INH ‐ isoniazid, RIF ‐ rifampicin; CIP ‐ ciprofloxacin; n/a ‐ not available. Log*P* computed by ChemDraw v22.2.0.

**Figure 6:**
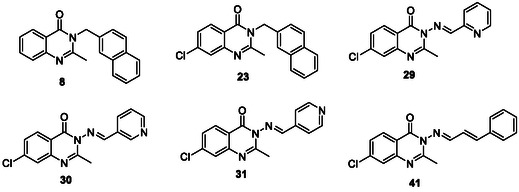
Structure of compounds **8**, **23**, **29**‐**31,** and **41**.

In series A, antimycobacterial activity was absent except for compound **6** bearing 3‐Cl, which exhibited activity against three different strains. Antimycobacterial activity was detected for some compounds belonging to series B, confirming the positive influence of chlorine atom at position 7. In series B, we found that the position of the substituents plays a crucial role; almost all compounds were substituted at the *para* position (4‐OCH_3_, 4‐F, 4‐CF_3_), compound **24** had a 3,4‐di substituent, and finally compound **27** featured chlorine at *meta* position. Interestingly, compound **14** (R = 4‐OH) was the only compound from the series with a *para* substitution that did not exert antimycobacterial activity. This may be due to the polar hydroxy group occupying that position while all the others have bulkier and more lipophilic substituents. Concerning series C, solubility in testing medium was a real challenge and limited in vitro evaluations. Using cosolvents did not improve the solubility, and salt formation for such structures was not an option. Yet the only compounds with sufficient solubility, namely compounds **25** and **32**, both with hydrophilic substituents, exerted antimycobacterial activity.

We have seven matching pairs among general structures A and B bearing the same substituents, while four pairs of matching pairs among the three structural types. Yet again due to the solubility issue within group C, we did not consider them for direct comparisons. Table S5, Supporting Information, shows the matching pairs with their antimycobacterial activity for direct comparisons. We see that in three cases, R = 4‐CH_3_, 4‐CF_3_, and 3,4‐diCl, compounds belonging to series B were more active when compared to their corresponding pair from series A. Again, compound **6** from series A (R = 3‐Cl) stands as an exception being more active than its series B matching compound.

Selected compounds with notable antimycobacterial activity (cut‐off value above), namely compounds **2, 6, 17, 19, 21, 24, 25, 32**, were further advanced to in vitro antimycobacterial activity evaluation against multidrug‐resistant (MDR) isolates of *Mtb*. For results, see **Table** **2** and refer to **Table** **3** for the resistance pattern of the isolates. All the tested compounds retained at least part of their potency against resistant strains. Interestingly, compound **17** showed a broad‐spectrum activity against all resistant strains. Taking into account its potent activity against *Mtb* H37Ra (MIC = 7.81 μg mL^−1^), *M. smeg*. (MIC = 31.25 μg mL^−1^), and *M. aurum* (MIC = 15.625 μg mL^−1^), compound **17** has the most extended spectrum of activity. The fact that the compound preserved activity against resistant isolates implies a diverse mechanism of action compared to employed standards for the susceptibility/resistance pattern.

**Table 2:** Antimycobacterial activity of selected compounds expressed as MICs against drug‐resistant clinical isolates. The values are in μg mL^−1^. INH: isoniazid.

	**Compound**							
Strain	**INH**	**6**	**17**	**19**	**21**	**24**	**25**	**32**
***Mtb IZAK***	>100	>100	**12.5**	>50	>100	>100	>25	**25**
***Mtb MATI***	>100	50	**12.5**	>50	>100	>100	**12.5**	**25**
***Mtb SORO***	>100	**25**	**12.5**	>50	>100	100	**12.5**	50
***Mtb TIAS***	>100	**25**	**12.5**	**12.5**	**12.5**	**6.25**	**25**	**12.5**
***Mtb YAGY***	>100	**12.5**	**12.5**	**12.5**	**12.5**	**6.25**	**6.25**	**25**
***Mtb TURZ***	>100	**25**	**12.5**	**12.5**	**12.5**	**6.25**	**25**	**25**
***Mtb*** **H37Rv**	0.2	50	**12.5**	**25**	**25**	50	**12.5**	**25**

**Table 3:** Susceptibility/resistance pattern of *Mtb* clinical isolates. The MIC values are in μg mL^−1^. R stands for resistant and S for susceptible.

Clinical strain	Resistance profile according to WHOa)	isoniazid	rifampicin	streptomycin	ethambutol	pyrazinamide	amikacin	moxifloxacin	clofazimine
***Mtb IZAK***	MDR	4 R	>8 R	4 R	0.5 S	>16 R	n.a.	n.a.	n.a.
***Mtb MATI***	MDR	>8 R	>8 R	>16 R	0.5 S	>128 R	n.a.	n.a.	n.a.
***Mtb SORO***	n.d.	4 R	0.125 S	4 R	2 R	Sb)	1 S	0.25 S	0.25 S
***Mtb TIAS***	n.d.	>8 R	0.125 S	4 R	1 R	Sb)	1 S	0.25 S	n.a.
***Mtb YAGY***	MDR	4 R	8 R	>16 R	4 R	Rb)	>64 R	0.25 S	n.a.
***Mtb TURZ***	MDR	>8 R	>8 R	16 R	1 R	Sb)	0,5 S	0.25 S	n.a.

a) MDR, Mtb resistant to rifampicin and isoniazid.^[^ ^31^ ^]^

b) Tested in mycobacteria growth indicator tube (MGIT) in concentration 100 mg/l. n.a. – not available.

### 3.2 Evaluation of in vitro Cytotoxicity

The compounds exhibiting the most promising antimycobacterial activity, namely compounds **2, 6, 17, 19, 21, 24, 25, 32,** were advanced to in vitro cytotoxicity evaluation in the hepatocellular cancer cell line HepG2 and MRC‐5 lung fibroblast cell line. The solubility of the compounds was the primary limiting factor for this testing. Data for the cytotoxic activity were evaluated after 24 h of incubation and, whenever possible, after 72 h of incubation. Only compounds **2** and **6** exhibited measurable toxicity, whereas the IC_50_ value of the other compounds was above the maximum achieved concentration (**Table** **4**). The cell viability curves (Figure S2, Supporting Information) and the methodology are located in the Supplementary material.

**Table 4:** In vitro cytotoxicity of selected compounds in HepG2 and MRC‐5 cel line.

Compound	IC_50_ for HepG2 [24 h] [μM]	IC_50_ for HepG2 [72 h]	IC_50_ for MRC‐5 [24 h] [μM]	IC_50_ for MRC‐5 [72 h]
**2**	305 ± 3	135 ± 6 μM	>300	215 ± 24 μM
**6**	507 ± 48	n.d.	551 ± 19	n.d.
**17**	>50	>50 μM	>100	>100 μM
**19**	>50	>50 μM	>100	>100 μM
**21**	>50	>50 μM	>50	>50 μM
**24**	>50	>50 μM	>50	>50 μM
**25**	>1000	n.d.	>1000	n.d.
**32**	>500	n.d.	>500	n.d.

n.d. – not determined

### 3.3 Target Proposal

Besides the obvious choice for the target, PBP, which comes from the design rationale of this study, we identified several other potential mycobacterial targets for our quinazolinone derivatives. The targets were identified through structure‐similarity searches of similar ligands (for methodology, see Supplementary Materials, section 1.9) against the Protein Data Bank (PDB) and ChEMBL databases, using representative general structures of series A, B, and C as input queries. We identified the following antimycobacterial targets: Protein recombinase A, RecA (UniProt ID: P9WHJ3) and replicative DNA helicase B, DnaB (UniProt ID: P9WMR3). For detailed results, see Supplementary Materials, section 2.7.

Nevertheless, in line with the original design of the series, these additional targets were not pursued in our study, which remained focused on the PBP proteins.

#### 3.3.1 PBP of Mycobacterium Tuberculosis

PBPs in *Mtb* are generally divided into two main groups: high‐molecular‐weight PBPs (HMW) and low–molecular‐weight PBPs (LMW). HMW PBPs are the enzymes involved in synthesizing and crosslinking the peptidoglycan layers. LMW PBPs are not directly involved in cell wall synthesis but participate in remodeling and maintenance of the cell wall.^[^ ^26^ ^]^ Our interest is focused primarily on the HMW PBPs. This group can be further divided into class A (ponA1, ponA2), which exhibits transglycosylase and transpeptidase activity, and class B (PBPa, PBPb), which only has transpeptidase activity. Specific roles and functions of individual PBP are still not entirely known. We focused our efforts on Class A since they possess both transglycosylase and transpeptidase activity, and targeting such bifunctional enzymes might have a greater impact on the pathogen's viability.^[^ ^27^ ^]^ PonA1 was designated to be essential for cell wall formation, which makes it a valuable target for antimycobacterial drugs.^[^ ^28^ ^]^ PonA2 could act as a β–lactamase,^[^ ^29^ ^]^ adding to the generally decreased sensitivity of mycobacteria to β–lactams.

#### 3.3.2 Docking

PBP 1 A–PonA1 (UniProt ID: P71707) was chosen for our docking studies. Generally, this protein consists of a transglycosylase and a transpeptidase domain and two disordered regions with a total length of 820 amino acids. There are two available crystallographic structures of PonA1 (pdb id: 5CRF, 5CXW), but both structures have only one domain, the transpeptidase domain important for the binding of β‐lactams. Inspired by the reported quinazolinone derivative (Figure 4), which is an allosteric modulator of PBP2 of S. aureus,^[^ ^21^ ^]^ we wanted to target potential allosteric sites of PonA1 rather than constraining the docking efforts to the known catalytic sites. Therefore, we used the complete structure of the AlphaFold homology model. The experimental structure (pdb id: 5CXW) was superposed with the AlphaFold model with RMSD = 0.446 Å (over the backbone of the transpeptidase domain), see Figure S3, Supporting Information.

**Figure 7:**
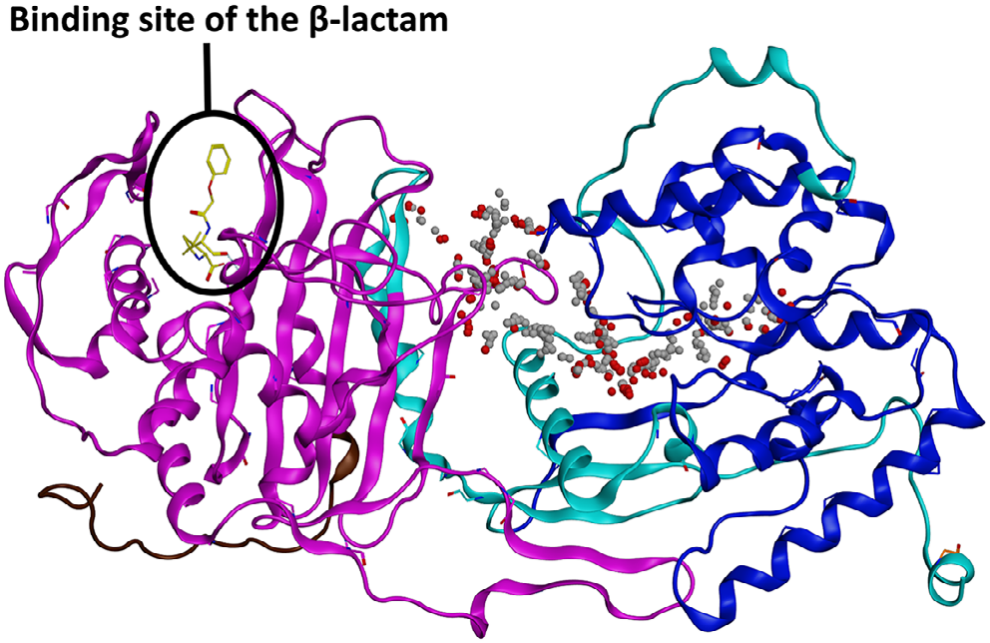
AlphaFold model of mycobacterial PonA1 (UniProt ID: P71707) with the transpeptidase domain (magenta), the transglycosylase domain (dark blue), and the connecting region (turquoise) penicillin‐binding site with bound β‐lactam (inferred from the overlay with the experimental structure pdb id: 5CXW) are highlighted in a black circle. The potential binding site created by SiteFinder is visualized with alpha centers (red dots represent potential polar interactions, gray dots represent potential lipophilic interactions).

We used Site Finder (MOE built‐in functionality, determining possible binding sites based on the topology and interaction potential) to identify potential binding sites. The candidate binding site consists of two best‐scored binding sites (see the scoring in Table S4 and two separate binding sites in Figure S1, Supporting Information). The combined binding site is indicated on the interface of the domains (see **Figure** **7**). Selected compounds with significant antimycobacterial activity from series B (compound **17**, R = 4‐OCH_3_) and C (compound **32**, R = 2,3‐diOH) were used for the docking studies. The docking was performed on rigid receptors, saving 10 best poses. Based on the docking score and observed interactions, we selected the best pose for each ligand with the most interactions between the receptor and quinazolinone core (hydrogen bond either with carbonyl group or nitrogen). The resulting docking poses of compounds 17 and 32 are located in Supplementary Material, Figure S4, Supporting Information, and were used to construct the systems for molecular dynamics simulations.

#### 3.3.3 Molecular Dynamics

The systems were solvated, minimized, heated, and equilibrated for a total time of 12 ns. Five independent 100 ns runs were performed for both systems in NAMD under AMBER10:EHT force field in the NPT ensemble at 101 kPa and 300 K. Based on the obtained RMSD values, both ligands **17** and **32** were stable (**Table** **5**).

**Table 5:** RMSD (Å) of heavy atoms of ligands averaged over the 100 ns trajectory.

	Replica					Avarage of replicas
	1	2	3	4	5	
Compound 17	1.706 ± 0.351	1.382 ± 0.531	1.895 ± 0.400	2.211 ± 0.484	1.880 ± 0.650	1.815 ± 0.494
Compound 32	1.459 ± 0.422	1.272 ± 0.254	1.628 ± 0.411	2.023 ± 0.565	1.681 ± 0.556	1.613 ± 0.556

*Trajectories aligned at receptor backbone atoms (C, Cα, N) average structure of the 100 ns trajectory*.

Compound **17** was stable during the whole 100 ns runs in each replica. The most abundant interaction was the H‐bond between the backbone of ARG399 (HBD) and the carbonyl oxygen of the quinazolinone core (HBA). The interaction was very strong, ranging up to −8.1 kcal mol^−1^ (as estimated by the force field), and could be observed in 22–98% of frames through all five replicas. The quinazolinone core could be additionally stabilized through H‐bond with ARG301 and N1 of the quinazolinone core. This interaction was, however, present only in two replicas in 16.75% or 22.51% of frames, respectively.

Compound **32** achieved a slightly shifted position after the docking compared to compound **17**. But also in this case, the pose was very stable in the following 100 ns MD simulation. The most abundant interaction was the H‐bond between the backbone of ASP477 (HBD) and the carbonyl oxygen of the quinazolinone core (HBA). This interaction could be observed in the range 64–93% in 4 replicas of 5. In the 5th replica, interaction to ASP477 was replaced with interaction to ARG399, which was not so often present in other runs. Other significant interactions were the H‐bonds between hydroxyl group of compound **32** and ASP340 or ASN474 in up to 99.84% frames, indicating a very favorable interaction. In two replicas, the quinazolinone core could be stabilized through the π–H interaction with VAL343 present in up to 20.91% frames.

**Figure 8:**
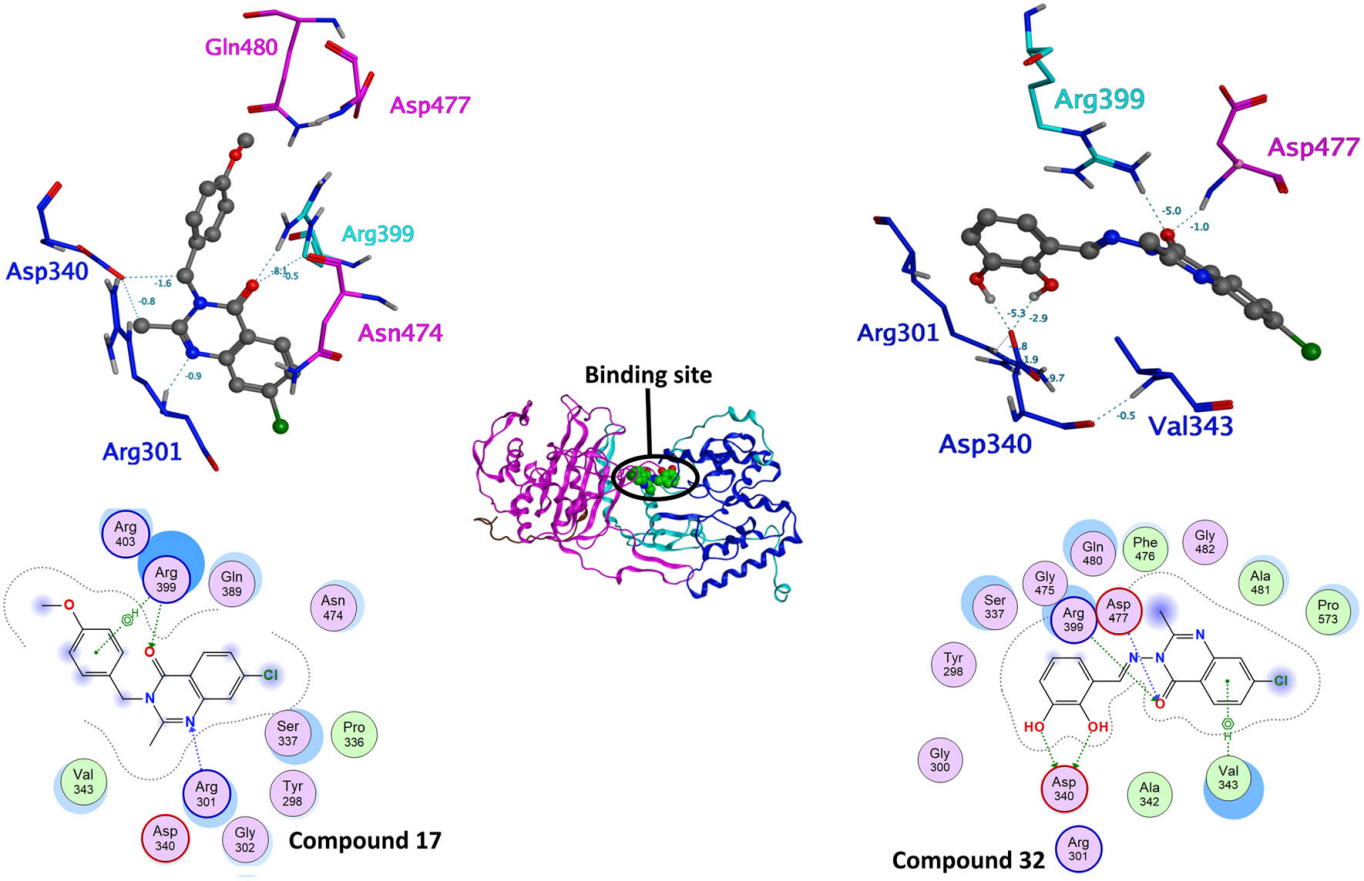
Representative binding modes of compounds **17** and **32** in mycobacterial PonA1 as predicted by molecular dynamics. Top: 3D visualization with the force field estimated energies of the interactions (kcal mol^−1^). Residues colored based on the domain. Bottom: 2D ligand–protein interaction diagrams. Center: depiction of the binding side between the domains.

Representative binding modes of compounds **17** and **32** from the MD simulations are depicted in **Figure** **8**. RMSD plots (ligands and protein, Figure S5–S8, Supporting Information) and detailed interaction occupancies (Table S6 and S7) are located in Supplementary Material.

### 3.4 Antibacterial Activity

In a microdilution in vitro assay, the final compounds were screened against four Gram‐positive [*Staphylococcus aureus*, MRSA, *Staphylococcus epidermidis*, *Enterococcus faecalis*] and four Gram‐negative [*Escherichia coli*, *Klebsiella pneumoniae*, *Acinetobacter baumannii*, *Pseudomonas aeruginosa*] bacterial strains of clinical importance. Antibacterial activity was expressed as MIC in μM read after 24 and 48 h of incubation. None of the tested compounds exerted significant activity up to the highest tested concentration, which was 500 μM for compounds with sufficient solubility in the testing medium.

### 3.5 Antifungal Activity

In a microdilution in vitro assay, the final compounds were screened against eight fungal strains [*Candida albicans*, *Candida krusei*, *Candida parapsilosis*, *Candida tropicalis*, *Aspergillus fumigatus*, *Aspergillus flavus* (AFla), *Lichtheimia corymbifera*, *Trichophyton interdigitale*] of clinical importance. Antifungal activity was expressed as MIC in μM read after 24 and 48 h of incubation (72 and 120 h for TI). None of the tested compounds exerted significant antifungal activity up to the highest tested concentration, which was 500 μM for compounds with sufficient solubility in the testing medium.

### 3.6 Final Considerations

We selected compounds **17**, **19**, **25,** and **32** as the most promising from our series. Compounds **17** and **19** belong to series B, and compounds **25** and **32** belong to series C. Best activity was observed in compound **17**, which proved decent activity against *Mtb* H37Ra, *Mtb* H37Rv, and MDR isolates of Mtb. The activity was consistent against all tested strains. Due to the limited solubility in the cultivation medium, the exact IC_50_ value representing toxicity on eukaryotic cells could not be determined. In series C, the solubility of the compounds seemed to be a determining aspect of their activity. Compounds **25** and **32**, containing hydroxy substituents on the benzene rings, posed a good compromise between the activity and sufficient solubility. These compounds were also nontoxic for human cell lines up to 1000 μM or 500 μM, respectively. None of the final compounds was active against tested bacterial or fungal strains. The summary of antimicrobial activities, in vitro toxicity, and druglikeness is shown in **Table** **6**. Comprehensive predicted ADMETox properties predicted by SwissADME are located in^[^ ^30^ ^]^ Table S7, Supporting Information.

**Table 6:** Resume of the antimicrobial activity, in vitro cytotoxicity, and druglikeness of the most promising compounds.

No.	MIC [μM]a)					IC_50_ [μM]		
	*Mtb* H37Ra	*Mtb* H37Rv	MDR *Mtb* H37Rv	Bacteria, incl. SA	Fungi	HepG2 24 h	MRC‐5 24 h	Druglike (Ro5)b)
**17**	25	40	40	inactive	inactive	>50	>100	Yes; 0 violation
**19**	26	83	41–165	inactive	inactive	>50	>100	Yes; 0 violation
**25**	87	35	17–70	inactive	inactive	>1000	>1000	Yes; 0 violation
**32**	47	76	38–152	inactive	inactive	>500	>500	Yes; 0 violation

a) MIC [μM] calculated from the measured MIC in mass concentration units [μg mL]^−1^.

b) compliance to Lipinski Rule of Five (Ro5), calculated by SwissADME.^[^ ^30^ ^]^ SA, *Staphylococcus aureus*; HepG2, hepatocellular cancer cell line; MRC‐5, lung fibroblast cell line.

## 4 Conclusions

This work explores the potential of a novel series of quinazolinone derivatives as antimycobacterial compounds. Among the 53 synthesized compounds from three different structural types, several demonstrated selective antimycobacterial activity with MIC values as low as 6.25 μg mL^−1^. Importantly, the activity was preserved in MDR clinical isolates of Mtb. Despite solubility challenges, particularly with series C, the results emphasize the importance of structural modifications, such as the inclusion of a chlorine atom at position 7 of the quinazolinone core, to enhance activity. Compound **17** emerged as a lead candidate, exhibiting broad‐spectrum and consistent antimycobacterial activity, including MDR strains. Docking and molecular dynamics simulations suggested stable binding of selected compounds (**17** and **32**) to mycobacterial PBP 1 A (PonA1, UniProt ID: P71707), occupying a potential allosteric site located in between the transglycosylase and transpeptidase domains. The key stabilizing interaction was the H‐bond between the carbonyl oxygen of the ligand and either ARG399 or ASP474 of the protein. The in silico simulations confirmed that PonA1 of Mtb is a valid target for our compounds. Although the design of the compounds was strongly inspired by antistaphylococcal compounds, the antibacterial activity was not observed among the compounds. The absence of significant antibacterial and antifungal activity in the tested compounds suggests a promising selectivity profile for antimycobacterial applications.

Future work will focus on optimizing water solubility, experimental confirmation of the target, and exploring synergism with existing anti(myco)bacterial drugs in order to advance these compounds as novel antitubercular drug candidates.

## Conflict of Interest

The authors declare no conflict of inteest

## Supporting information

Supplementary Material

## Data Availability

The data that support the findings of this study are available from the corresponding author upon reasonable request.
