# Supplementary material for: Novel Quinazolinones Active against Multidrug‐Resistant Mycobacterium Tuberculosis: Synthesis, Antimicrobial Evaluation, and in Silico Exploration of Penicillin‐Binding Protein 1A as a Potential Target
Source: ChemMedChem. 2025 Jun 8;20(13):e202500147. doi: 10.1002/cmdc.202500147 (PMC12221116; doi:10.1002/cmdc.202500147)
Supplement: Supplementary file 1 — Supplementary Material [file CMDC-20-e202500147-s001.pdf]

# Supplementary materials

1) Methods ........ 2

1.1) *In vitro* screening of antimycobacterial activity against *M. tuberculosis* H37Ra, *M. smegmatis*, *M. aurum*, *M. avium* ........ 2

1.2) *In vitro* screening of antimycobacterial activity against *M. tuberculosis* H37Rv and *M. kansasii* ........ 3

1.3) *In vitro* screening of antimycobacterial activity against drug-resistant clinical isolates of *Mycobacterium tuberculosis* ........ 4

1.4) *In vitro* antibacterial evaluation ........ 4

1.5) *In vitro* antifungal evaluation ........ 5

1.6) Cytotoxicity evaluation ........ 7

1.7) Crystallographic confirmation of the double bond configuration ........ 7

1.8) Identification of the binding site of PonA1 ........ 8

1.9) Similarity searching ........ 8

2) Results ........ 10

2.1) Characterization of compounds and analytical data ........ 10

2.2) Results of antimycobacterial activity ........ 19

2.3) Results of cytotoxicity evaluation ........ 20

2.4) Results of in silico experiments ........ 21

2.4.1) Docking ........ 21

2.4.2) Molecular dynamics – compound 17 ........ 22

2.4.3) Molecular dynamics – compound 32 ........ 23

2.5) Crystallographic confirmation of double bond configuration ........ 24

2.6) *In silico* prediction of pharmacokinetic properties of selected compounds ........ 26

2.7) Results of the similarity searching ........ 27

References ........ 28

# 1) Methods

## 1.1) *In vitro* screening of antimycobacterial activity against *M. tuberculosis* H37Ra, *M. smegmatis*, *M. aurum*, *M. avium*

The antimycobacterial assay was performed with rapidly growing *Mycolicibacterium smegmatis* DSM 43465 (ATCC 607), *Mycolicibacterium aurum* DSM 43999 (ATCC 23366), and non-tuberculous mycobacterial species *Mycobacterium avium* DSM 44156 (ATCC 25291). These strains were obtained from the German Collection of Microorganisms and Cell Cultures (Braunschweig, Germany). The avirulent strain of *Mycobacterium tuberculosis* H37Ra (*Mtb*) ITM-M006710 (ATCC 9431) was obtained from the Belgian Coordinated Collections of Microorganisms (Antwerp, Belgium). The technique used for activity determination was the microdilution broth panel method using 96-well microtitration plates. The culture medium was Middlebrook 7H9 broth (Merck, Darmstadt, Germany) enriched with 0.4% glycerol (Merck, Darmstadt, Germany) and 10% Middlebrook OADC growth supplement (Himedia, Mumbai, India).[1]

The mycobacterial strains were cultivated on supplemented Middlebrook 7H9 agar, and suspensions were prepared in supplemented Middlebrook 7H9 broth. The final density was adjusted to 1.0 on the McFarland scale and diluted in either 1:20 (for rapidly growing mycobacteria) or 1:10 (for slow-growing mycobacteria) with broth.

The tested compounds were dissolved in DMSO (Merck, Darmstadt, Germany), and then Middlebrook broth was added to obtain a concentration of 2000 µg/mL. The standards used for activity determination were isoniazid (INH), rifampicin (RIF), and ciprofloxacin (CIP) (Merck, Darmstadt, Germany). Final concentrations were reached by binary dilution with broth and adding mycobacterial suspension. The final concentrations were set as 500, 250, 125, 62.5, 31.25, 15.625, 7.81, and 3.91 µg/mL. Isoniazid was diluted in the range 500–3.91 µg/mL for screening against rapidly growing mycobacteria, 2000–15.625 µg/mL for *M. avium*, and 1–0.0078 µg/mL for *Mtb*. RIF final concentrations ranged from 50 to 0.39 µg/mL for rapidly growing mycobacteria, from 1 to 0.0078 µg/mL for *M. avium*, and from 0.1 to 0.00078 µg/mL for *Mtb*. CIP was used for screening antimycobacterial activity with final concentrations of 1, 0.5, 0.25, 0.125, 0.0625, 0.0313, 0.0156, 0.0078 µg/mL. The final concentration of DMSO did not exceed 2.5% (v/v) and did not affect the growth of all strains. Positive (broth, DMSO, bacteria) and negative (broth, DMSO) growth controls were included.

Plates containing slow-growing mycobacteria were sealed with polyester adhesive film and all plates were incubated in the dark at 37°C without agitation. A 0.01% solution of resazurin sodium salt was added after 48 hours of incubation for *M. smegmatis*, after 72 hours for *M. aurum*, after 96 hours for *M. avium*, and after 120 hours for *Mtb*. Microtitration plates were then incubated for 2.5 hours to determine the activity against *M. smegmatis*, 4 hours for *M. aurum*, 6 hours for *M. avium*, and 18 hours for *Mtb*.

The antimycobacterial activity was expressed as minimum inhibitory concentration (MIC) determined based on the stain colour change (blue colour – no growth, active compound/concentration; pink colour – positive growth, inactive compound/concentration (for MIC of standards, see Table S1). All experiments were conducted in duplicates.

Table S1: Results of internal quality controls (standards) in antimycobacterial screening.

| | **MINIMUM INHIBITORY CONCENTRATION (µg/mL)** | | |
|---|---|---|---|
| **MYCOBACTERIAL STRAIN** | Ciprofloxacin | Rifampicin | Isoniazid |
| *Mycolicibacterium smegmatis* DSM 43465 (ATCC 607) | 0.0625–0.125 | 12.5–25 | 15.625–31.25 |
| *Mycolicibacterium aurum* DSM 43999 (ATCC 23366) | 0.0078–0.0156 | 0.39–0.78 | 1.95–3.91 |
| *Mycobacterium avium* DSM 44156 (ATCC 25291) | 0.5 | 0.0625–0.125 | 500–1000 |
| *Mycobacterium tuberculosis* H37Ra ITM-M006710 (ATCC 9431) | 0.125–0.25 | 0.00156–0.0031 | 0.125–0.25 |

## 1.2) *In vitro* screening of antimycobacterial activity against *M. tuberculosis* H37Rv and *M. kansasii*

Microdilution method based on Microplate Alamar Blue Assay (MABA)[2,3] was applied. Tested strain *Mycobacterium tuberculosis* H37Rv CNCTC My 331/88 (ATCC 27294) was obtained from the Czech National Collection of Type Cultures (CNCTC), National Institute of Public Health (Prague, Czech Republic). *Mycobacterium kansasii* DSM 44162 (ATCC 12478) was obtained from the German Collection of Microorganisms and Cell Cultures (Braunschweig, Germany). Middlebrook 7H9 broth of declared pH 6.6 (Sigma-Aldrich) enriched with 0.4% of glycerol (Sigma-Aldrich) and 10% of OADC growth supplement (Himedia, Mumbai, India) was used for cultivation.

Tested compounds were dissolved and diluted in DMSO and mixed with broth (25 µL of DMSO solution in 2.475 mL of broth) and placed (100 µL) into microplate wells. Mycobacterial inocula were prepared in isotonic saline solution and the density was adjusted to 0.5–1.0 according to McFarland scale. These suspensions were diluted by $10^{-1}$ and used to inoculate the testing wells, adding 100 µL of mycobacterial suspension per well. The final concentrations of tested compounds in wells were 100, 50, 25, 12.5, 6.25, 3.13 and 1.56 µg/mL. Isoniazid (INH) was used as a standard (inhibition of growth). Positive control (visible growth) consisted of broth plus mycobacterial suspension plus DMSO. A total of 30 µL of Alamar Blue working solution (1:1 mixture of 0.02% resazurin sodium salt (aq. sol.) and 10% Tween 80) was added after five days of incubation. Results

were then determined after 24 h of incubation. The MIC (in μg/mL) was defined as the lowest concentration that prevented the blue-to-pink colour change. All experiments were conducted in duplicates.

## 1.3) *In vitro* screening of antimycobacterial activity against drug-resistant clinical isolates of *Mycobacterium tuberculosis*

The isolates of drug-resistant *M. tuberculosis* were gathered in Faculty Hospital in Hradec Králové. *Mtb* laboratory ID IZAK was isolated from a bronchial aspirate of a 63-year-old man in 2020, *Mtb* laboratory ID MATI was isolated from the sputum of a 23-year-old man, *Mtb* laboratory ID SORO was isolated from the sputum of a 40-year-old woman, *Mtb* laboratory ID TIAS was isolated from the sputum of a 25-year-old man, *Mtb* laboratory ID YAGY was isolated from the bronchial aspirate of a 48-year-old man, and *Mtb* laboratory ID TURZ was isolated from a 46-year-old woman. The susceptibility profile (Table 4 in the main article) was determined according to the methodology defined by Clinical and Laboratory Standard Institute.[4]

## 1.4) *In vitro* antibacterial evaluation

The microdilution broth method was performed according to EUCAST (The European Committee on Antimicrobial Susceptibility Testing) instructions with slight modifications.[5] Eight tested bacterial strains were purchased from the Czech Collection of Microorganisms (CCM, Brno, Czech Republic) or from the German Collection of Microorganisms and Cell Cultures (DSM, Braunschweig, Germany): *Staphylococcus aureus* subsp. *aureus* CCM 4223 (ATCC 29213), *Staphylococcus aureus* subsp. *aureus* methicillin-resistant (MRSA) CCM 4750 (ATCC 43300), *Staphylococcus epidermidis* CCM 4418 (ATCC 12228), *Enterococcus faecalis* CCM 4224 (ATCC 29212), *Escherichia coli* CCM 3954 (ATCC 25922), *Klebsiella pneumoniae* CCM 4415 (ATCC 10031), *Acinetobacter baumannii* DSM 30007, ATCC 19606, *Pseudomonas aeruginosa* CCM 3955 (ATCC 27853). The cultivation was done in Cation-adjusted Mueller-Hinton broth (CAMHB, M-H 2 Broth, Merck) at 35±2 °C. Tested compounds were dissolved in DMSO (Merck) to produce stock solutions. The final concentration of DMSO in the cultivation medium did not exceed 1% (v/v) of the total solution composition and did not affect the growth of bacteria. Positive controls consisted of test microbe, cultivation medium and DMSO, while negative controls consisted of cultivation medium and DMSO. Antibacterial activity was expressed as minimum inhibitory concentration (MIC, in μM) after 24 and 48 h of static incubation in the dark and humidified atmosphere at 35±2 °C. Visual inspection and spectrophotometric detection were used for MIC endpoint evaluation. The internal quality standards gentamicin and ciprofloxacin (both from Merck) were involved in assays (for MIC of standards, see Table S2).

Table S2: Results of internal quality controls (standards) in antibacterial screening.

| | MINIMUM INHIBITORY CONCENTRATION | | | |
|---|---|---|---|---|
| | ciprofloxacin (μg/mL) | | gentamicin (μg/mL) | |
| BACTERIAL STRAIN | spectrophotometric detection* | visual detection** | spectrophotometric detection* | visual detection** |
| *Staphylococcus aureus* subsp. *aureus* ATCC 29213, CCM 4223 | 0.128 | 0.128-0.256 | 1 | 1 |
| *Staphylococcus aureus* subsp. *aureus*, MRSA ATCC 43300, CCM 4750 | 0.128 | 0.128 | 16–32 | 16–32 |
| *Staphylococcus epidermidis* ATCC 12228, CCM 4418 | >1,024 | >1,024 | >8 | >8 |
| *Enterococcus faecalis* ATCC 29212, CCM 4224 | 0.512 | 0.512 | 16 | 8 |
| *Escherichia coli* ATCC 25922, CCM 3954 | 0.008 | 0.008 | 1–2 | 1–2 |
| *Klebsiella pneumoniae* ATCC 10031, CCM 4415 | >1,024 | >1,024 | >8 | >8 |
| *Acinetobacter baumannii* ATCC 19606, DSM 30007 | 0.256 | 0.256 | 2 | 2 |
| *Pseudomonas aeruginosa* ATCC 27853, CCM 3955 | 0.128 | 0.128 | 0.5 | 0.5 |

*The MIC of antibacterial agents is the lowest concentration giving rise to an inhibition of growth of 95% of that of the drug-free control. Results were read after 24 h microdilution plates cultivation without agitation at 35±2 °C in a humidified atmosphere. Measure on a microplate reader (SynergyTM HTX, BioTek Instruments, Inc., USA) at wavelength 530 nm.
**The MIC was determined by naked eye in the well with the lowest drug concentration, where no visible growth of microbial agent was detected. Results were read after 24 h incubation without agitation at 35±2 °C in a humidified atmosphere.

## 1.5) *In vitro* antifungal evaluation

Antifungal activity evaluation was performed using a microdilution broth method according to EUCAST instructions (The European Committee on Antimicrobial Susceptibility Testing) with slight modifications.[6] Eight fungal strains (four yeast and four mould strains) were used for antifungal activity screening, namely: *Candida albicans* CCM 8320 (ATCC 24433), *Candida krusei* CCM 8271 (ATCC 6258), *Candida parapsilosis* CCM 8260 (ATCC 22019), *Candida tropicalis* CCM 8264 (ATCC 750), *Aspergillus fumigatus* ATCC 204305, *Aspergillus flavus* CCM 8363, *Lichtheimia corymbifera* CCM 8077, and *Trichophyton interdigitale* CCM 8377 (ATCC 9533). Testing strains were purchased from the Czech Collection of Microorganisms (CCM, Brno, Czech Republic) or from the American Type Collection Cultures (ATCC, Manassas, VA, USA). Tested compounds were dissolved in DMSO and diluted in a twofold serial dilution with RPMI 1640 medium, with glutamine and 2% glucose (Merck), buffered to pH 7.0 with MOPS (3-morpholinopropane-1-sulfonic acid, Merck). The final

concentration of DMSO in the testing medium did not exceed 1% (v/v). Static incubation was performed in the dark in a humid atmosphere. The results were read after 24 h (yeasts) or 48 h (moulds) cultivation without agitation at 36.8 °C in a humidified atmosphere. *Trichophyton interdigitale* was cultivated for 120 h at 26.0 °C. Positive growth controls consisted of test microbe, cultivation medium and DMSO, while negative controls consisted of cultivation medium and DMSO. After static incubation in the dark, visual inspection and spectrophotometric detection were used for MIC endpoint evaluation. The internal quality standards, amphotericin B (Merck) and voriconazole (Toronto Research Chemicals, Inc., Toronto, ON, Canada), were involved in assays (for MIC of standards, see Table S3).

Table S3: Results of internal quality controls (standards) in antifungal screening

| | MINIMUM INHIBITORY CONCENTRATION | | | |
|---|---|---|---|---|
| | amphotericin B (µg/mL) | | voriconazole (µg/mL) | |
| YEAST/MOULD STRAIN | $IC_{90}$, spectrophotometric detection* | MIC, visual detection** | $IC_{50}$, spectrophotometric detection*** | MIC, visual detection** |
| *Candida albicans* ATCC 24433, CCM 8320 | 0.5 | 0.5 | 0.03 | >16 |
| *Candida krusei* ATCC 6258, CCM 8271 | 1 | 1 | 0.25 | 0.5 |
| *Candida parapsilosis* ATCC 22019, CCM 8260 | 0.5 | 0.5 | 0.03 | 8 |
| *Candida tropicalis* ATCC 750, CCM 8264 | 1 | 1 | 0.0625 | >16 |
| *Aspergillus fumigatus* ATCC 204305 | 1 | 1 | 0.5 | 1 |
| *Aspergillus flavus* CCM 8363 | 8 | 8 | 4 | >16 |
| *Lichtheimia corymbifera* CCM 8077 | 0.5 | 0.5 | >16 | >16 |
| *Trichophyton interdigitale* ATCC 9533, CCM 8377 | 2 | 2 | >16 | >16 |

*The $IC_{90}$ of amphotericin B is the lowest concentration giving rise to an inhibition of growth of 90% of that of the drug-free control. Results were read after 24 h (yeasts) or 48 h (moulds, except *Trichophyton interdigitale*) of cultivation without agitation at 36.8 °C in a humidified atmosphere and measured on a microplate reader (SynergyTM HTX, BioTek Instruments, Inc., USA) at wavelength 530 nm. *Trichophyton interdigitale* was cultivated for 120 h, at 26°C.

**The MIC (minimum inhibitory concentration) was determined by naked eye in the well with the lowest drug concentration, where no visible growth of microbial agent was detected. Results were read after 24 h (yeasts) or 48 h (moulds, except *Trichophyton interdigitale*) cultivation without agitation at 35±2 °C in a humidified atmosphere. *Trichophyton interdigitale* was cultivated for 120 h, at 26 °C.

***The $IC_{50}$ of azole (voriconazole) antifungal agents is the lowest drug concentration giving inhibition of growth of 50% compared to the drug-free control. Results were read after 24 h (yeasts) or 48 h (moulds, except *Trichophyton interdigitale*) cultivation without agitation at 35±2 °C in a humidified atmosphere. Measured on a microplate reader (SynergyTM HTX, BioTek Instruments, Inc., USA) at wavelength 530 nm. *Trichophyton interdigitale* was cultivated for 120 h, at 26 °C.

## 1.6) Cytotoxicity evaluation

MRC-5 (human normal lung fibroblasts) and Hep G2 (human liver hepatocellular carcinoma) cell lines were obtained from American Type Culture Collection (Manassas, Virginia, USA) and maintained in Dulbecco's Modified Eagle's Medium without phenol red (Capricorn Scientific GmbH, Ebsdorfergrund, Germany) supplemented with 10% (v/v) foetal bovine serum (Capricorn), 4 mM l-alanyl-l-glutamine (Capricorn), 10 mM HEPES buffer and 1× penicillin/streptomycin solution (stock solution consists of $10^7$ Units/L of penicillin G sodium and 10 000 mg/L streptomycin sulphate in saline; Capricorn). Medium for MRC-5 is further supplemented with 1× MEM non-essential amino acid solution (Sigma-Aldrich). Cells were sub-cultured every 3 to 4 days. Evaluation of cytotoxicity was performed on cells seeded on 96 well plates with flat bottom (TPP, Trasadingen, Switzerland) at the concentration 10 000 cells per well. Stock solutions of studied compounds were prepared in DMSO. Compounds were added 24 h after seeding in the concentration range from 1 to 1000 µM in cell culture medium and incubated with the cells for 24 or 72 h. Final concentration of DMSO in each experiment was kept under 1% (v/v) to prevent any DMSO toxicity, which was also assayed in each experiment. Cells were observed under routine microscope at the end of incubation (24 or 72 h) to assess approximate limit of solubility of studied compounds in cell culture medium (presence of crystals in cell culture; results with concentrations exceeding limit of solubility were not used in the assessment of cytotoxicity).

Cellular viability was assessed using neutral red (NR) uptake assay. Cell culture medium was replaced with medium containing 55 µg/mL of NR (Sigma-Aldrich) and cells were incubated with NR for 2 h. After incubation, cells were fixed for 15 min using 1% (w/v) $CaCl_2$ (anhydrous; Penta) in 0.5% formaldehyde (VWR/Avantor, Radnor, Pennsylvania, USA), washed twice with phosphate buffered saline (Sigma-Aldrich) and lysed 30 min with 1% (v/v) glacial acetic acid (Penta) in 50% (v/v) ethanol (Penta). Absorbance in each well of the plate was subsequently measured using Tecan Infinite M200 Pro plate reader (Tecan Group Ltd., Zürich, Switzerland) at $\lambda$ = 540 nm. Mean value of absorbance from the wells with cells treated with lethal dose of hydrogen peroxide was subtracted from each well. Results are expressed as % of untreated control cells (100%).

## 1.7) Crystallographic confirmation of the double bond configuration

Crystals of **47** were obtained at room temperature as colourless needles by the slow evaporation of a chloroform solution of the compound. X-ray intensity data were collected at 293(2) K with a Rigaku XtaLAB Synergy diffractometer (Rigaku, Tokyo, Japan), equipped with a microfocus PhotonJet Mo-Kα source and a Hybrid Pixel Array Detector. Data collection was performed with omega scans with a step size of 0.5° and an exposure time of 320 s/frame. A total of 11,337 Bragg reflections were collected, giving a metrically orthorhombic unit; the analysis of the systematic absences indicated the space group P212121. Intensity data were integrated and empirically corrected for Lorentz-polarization and absorption effects, using the Rigaku CrysAlisPro 1.171.43.95a software (Rigaku Oxford Diffraction/Agilent Technologies UK Ltd.). The structure was solved by direct methods using SIR2019/3[7] and completed by iterative cycles of full-matrix least-squares refinement on Fo2 and ΔF synthesis using SHELXL-2019/3[8] within the WinGX suite (WinGX v.2023.1).[9] The *E*-configuration of the double bond was unequivocally determined by the calculation of the Flack parameter, exploiting the anomalous dispersion effect provided by the chlorine atom. Hydrogen atoms were introduced at calculated positions in their described geometries and allowed to ride on the attached atom with fixed isotropic thermal parameters (1.2 and 1.5 Ueq of the parent atom for aromatic and methyl, respectively).

The structure was analysed with PARST[10], and the graphical representations were rendered with Mercury 2024.3.0.[11]

## 1.8) Identification of the binding site of PonA1

Table S4. Calculated values from SiteFinder MOE (Molecular Operating Environment, v2022.02).

| Site | Size | PLB | HYD | Side |
|---|---|---|---|---|
| 1 | 339 | 5.04 | 85 | 159 |
| 2 | 225 | 1.89 | 37 | 95 |
| 3 | 102 | 0.75 | 22 | 37 |
| 4 | 46 | 0.28 | 19 | 37 |

Size: number of alpha spheres comprising the site, PLB: Propensity for Ligand Binding – based on amino acids composition of the binding site, HYD: number of hydrophobic contact atoms, Side: sidechain contact atoms in the receptor

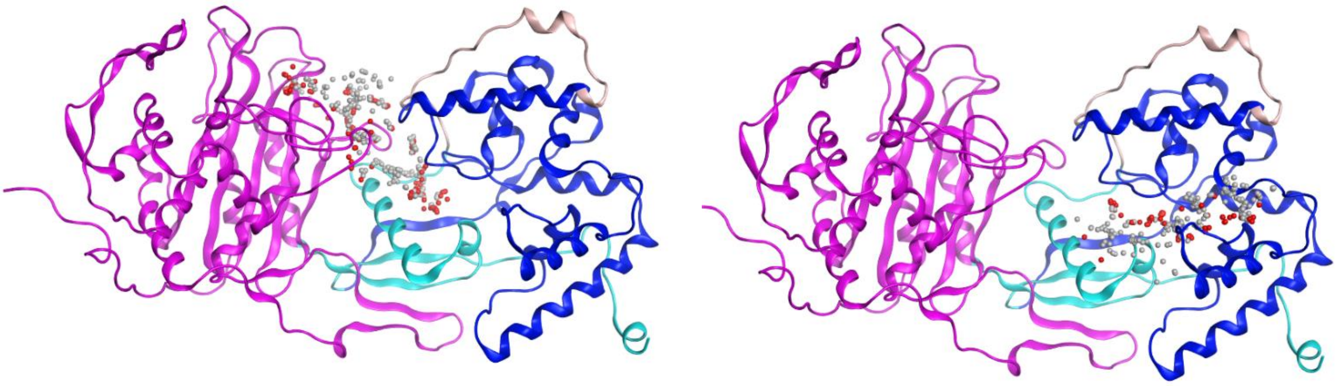

Figure S1 Alphacenters of binding site 1 on the left side and binding site 2 on the right side.

## 1.9) Similarity searching

**Database Preparation from the Protein Data Bank (PDB)**

A dataset of ligands with potential antimycobacterial activity was prepared from the Protein Data Bank (PDB) as of June 11, 2024. First, the PDB was filtered for entries with a source organism in the family *Mycobacteriaceae* (https://www.rcsb.org/search/browse/taxonomy) and containing "non-polymer" molecules. The initial dataset comprised 9,366 non-polymer entities (putative ligands), which were downloaded in CSV format, with ligands represented as SMILES strings. To ensure data quality, several preprocessing steps were applied. Entries identified as artifacts or corrupted were first removed. Then, a custom Python script was used to resolve formatting issues within the CSV file—specifically, newline characters (\n) erroneously present within double-quoted text fields. These characters were removed to preserve structural integrity during parsing. The Molecular Operating Environment (MOE, v2022.02; Chemical Computing Group, Ontario, Canada) was used to process the ligand database. Using the *Wash* utility, ligands were neutralized and 2D coordinates were generated. Basic molecular descriptors were then

calculated. Ligands with fewer than five heavy atoms or lacking any carbon atoms were excluded, yielding a final set of 5,205 entries (1,684 unique ligands). These filters ensured the retention of chemically relevant, organic small molecules associated with binding to mycobacterial proteins. Finally, the MACCS (166-bit) structural keys were calculated and stored in binary format for each ligand.

**Database Preparation from the ChEMBL database**

A second ligand dataset was prepared using the ChEMBL database (June 11, 2024), focusing on compounds associated with *Mycobacterium* targets. A search for “Mycobacterium” was performed across all target types, including whole organisms, proteins, and nucleic acids. All resulting targets were selected to ensure broad coverage of relevant biological systems. The associated bioactivity data were then accessed, yielding 531,891 activity records containing 115,037 unique ligands). The dataset was exported and successfully converted into a MOE database. Ligands were processed using the *Wash* utility in MOE to ensure standardization; although the compounds were already neutralized, desalting was performed to remove counterions and other non-relevant fragments. Finally, MACCS (166-bit) structural keys were calculated and stored in binary format for each ligand.

**Similarity Searching**

Simplified representatives of the individual series of the quinazolinones were selected and their MACCS (166-bit) structural keys were calculated. Using MOE, they were used to query the databases to fetch structurally similar ligands, using the criterion of Tanimoto coefficient (TI) > 0.85.

# 2) Results

## 2.1) Characterization of compounds and analytical data

Characterization of **intermediate I**: Light brown solid; Yield 94 %; LRMS (APCI) m/z: $[M-H]^-$ = 159.2, $[M+H]^+$ = 161.3 (exact mass = 161.05); $^1$H NMR (600 MHz, DMSO-*d₆*): δ 8.05 (ddd, 1H), 7.86 (ddd, 1H), 7.59 – 7.49 (m, 2H), 2.36 (s, 3H, $CH_3$).

Characterization of **intermediate II**: Brown solid; Yield 97%; LRMS (APCI) m/z: $[M-H]^-$ = 194.0, $[M+H]^+$ = 196.4 (exact mass = 195.01); $^1$H NMR (600 MHz, DMSO-$d_6$): δ 8.04 (d, J = 8.4 Hz, 1H), 7.61 (d, J = 2.1 Hz, 1H), 7.58 (dd, J = 8.4, 2.1 Hz, 1H), 2.36 (s, 3H, $CH_3$); $^{13}$C NMR (151 MHz, DMSO-$D_6$): δ 162.28, 159.08, 147.89, 141.75, 130.27, 128.98, 126.11, 116.02, 21.59.

Characterization of **intermediate III**: Brown solid; Yield 85 %; LRMS (APCI) m/z: $[M-H]^-$ = 208.0, $[M+H]^+$ = 210.3 (exact mass = 209.04); $^1$H NMR (DMSO-$d_6$): δ 7.47–7.52 (m, 1H), 7.36–7.40 (m, 1H), 6.39–6.45 (m, 2H), 5.41 (s, 2H, $NH_2$), 2.40 (s, 3H. $CH_3$).

**3-(2,4-Dimethoxybenzyl)-2-methylquinazolin-4(3*H*)-one** (**1**). White solid; Yield 23%; m.p. 180–182 °C; $^1$H NMR (600 MHz, DMSO-$d_6$): δ 8.10 – 8.06 (m, 1H), 7.80 – 7.74 (m, 1H), 7.58 (d, *J* = 8.0 Hz, 1H), 7.46 (t, *J* = 7.5 Hz, 1H), 6.62 – 6.57 (m, 2H), 6.41 – 6.36 (m, 1H), 5.15 (s, 2H, $CH_2$), 3.81 (s, 3H, $OCH_3$), 3.69 (s, 3H, $OCH_3$), 2.42 (s, 3H, $CH_3$); $^{13}$C NMR (151 MHz, DMSO-$d_6$): δ 161.94, 160.41, 157.86, 155.82, 147.69, 134.96, 127.14, 127.05, 126.90, 126.86, 120.43, 116.46, 105.45, 99.07, 56.13, 55.75, 42.35, 23.07; IR (ATR-Ge, $cm^{-1}$): 2996 (C–H stretch), 1673 (amidic C=O carbonyl stretch), 1590, 1568 (C–C aromatic stretch); Elemental analysis: Calculated for $C_{18}H_{18}N_2O_3$ (MW 310.35): 69.66% C; 5.85% H; 9.03% N. Found: 69.88% C; 5.63% H; 9.14% N.

**3-(3-Methoxybenzyl)-2-methylquinazolin-4(3*H*)-one (2).** White solid; Yield 30%; m.p. 179–180 °C; $^1$H NMR (600 MHz, DMSO-*$d_6$*): δ 8.14 – 8.09 (m, 1H), 7.81 – 7.75 (m, 1H), 7.58 (d, *J* = 8.1 Hz, 1H), 7.48 (t, *J* = 7.5 Hz, 1H), 7.21 (t, *J* = 7.9 Hz, 1H), 6.84 – 6.79 (m, 1H), 6.74 (t, *J* = 2.1 Hz, 1H), 6.66 (d, *J* = 7.5 Hz, 1H), 5.31 (s, 2H, $CH_2$), 3.68 (s, 3H, $OCH_3$), 2.45 (s, 3H, $CH_3$); $^{13}$C NMR (151 MHz, DMSO-*$d_6$*): δ 162.03, 160.11, 155.68, 147.66, 138.69, 135.07, 130.54, 127.18, 127.00, 126.97, 120.38, 118.66, 113.02, 112.91, 55.58, 46.80, 23.44; IR (ATR-Ge, $cm^{-1}$): 3065 (C–H stretch), 1674 (amidic C=O carbonyl stretch), 1599, 1571 (C–C aromatic stretch); Elemental analysis: Calculated for $C_{17}H_{16}N_2O_2$ (MW 280.33): 72.84% C; 5.75% H; 9.99% N. Found: 72.90% C; 5.69% H; 9.84% N.

**3-(4-Methoxybenzyl)-2-methylquinazolin-4(3*H*)-one (3).** White solid; Yield 38%; m.p. 177–179 °C; $^1$H NMR (600 MHz, DMSO-*$d_6$*): δ 8.12 – 8.08 (m, 1H), 7.83 – 7.77 (m, 1H), 7.61 (d, *J* = 7.9 Hz, 1H), 7.51 – 7.46 (m, 1H), 7.22 (d, *J* = 7.4 Hz, 1H), 7.16 – 7.10 (m, 1H), 7.08 – 7.02 (m, 1H), 6.52 (d, *J* = 7.7 Hz, 1H), 5.27 (s, 2H, $CH_2$ 130.50, 128.60, 127.34, 126.81, 126.70, 120.34, 114.12, 55.81, 45.30, 22.90; IR (ATR-Ge, $cm^{-1}$): 2996 (C–H stretch), 1669 (C=O carbonyl stretch), 1596, 1570, 1557 (C–C aromatic stretch); Elemental analysis: Calculated for $C_{17}H_{16}N_2O_2$ (MW 280.33): 72.84% C; 5.75% H; 9.99% N. Found: 72.62% C; 5.97% H; 9.88% N.

**3-(3-Fluorobenzyl)-2-methylquinazolin-4(3*H*)-one (4).** White solid; Yield 20%; m.p. 170–172 °C; $^1$H NMR (600 MHz, $CDCl_3$) 8.30 – 8.26 (m, 1H), 7.76 – 7.70 (m, 1H), 7.62 (d, *J* = 8.0 Hz, 1H), 7.45 (t, *J* = 7.4 Hz, 1H), 7.32 – 7.24 (m, 2H), 6.98

– 6.91 (m, 1H), 6.91 – 6.86 (m, 1H), 5.36 (s, 2H, $CH_2$), 2.52 (s, 3H, $CH_3$).; $^{13}C$ NMR (151 MHz, $CDCl_3$): δ 164.08, 162.45, 154.35, 147.43, 138.58, 134.66 (d, *J* = 32 Hz), 130.86, 130.66, 126.92, 126.79 (d *J* = 272.4 Hz), 122.18, 120.38, 114.92 (d, *J* = 6 Hz), 113.66 (d, *J* = 4 Hz), 46.80, 23.45; IR (ATR-Ge, $cm^{-1}$): 3057 (C–H stretch), 1671 (C=O carbonyl stretch), 1613, 1596, 1572 (C–C aromatic stretch).

**2-Methyl-3-(2-methylbenzyl)quinazolin-4(3*H*)-one (5).** Yellow solid; Yield 56%; m.p. 172–174 °C; $^1H$ NMR (600 MHz, $CDCl_3$): δ 8.28 (dd, *J* = 6.4, 0.6 Hz, 1H), 7.78 – 7.72 (m, 1H), 7.65 (d, *J* = 7.7 Hz, 1H), 7.48 – 7.44 (m, 1H), 7.16 (t, *J* = 8.0 Hz, 2H), 7.10 – 7.04 (m, 1H), 6.68 – 6.63 (m, 1H), 5.32 (s, 2H , $CH_2$), 2.48 (s, 3H, $CH_3$), 2.41 (s, 3H, $CH_3$); $^{13}C$ NMR (151 MHz, $CDCl_3$): δ 162.35, 154.80, 147.53, 134.55, 133.52, 132.80, 130.66, 128.66, 128.23, 127.25, 123.92, 122.75, 121.66, 120.38, 44.99, 23.13, 19.24; IR (ATR-Ge, $cm^{-1}$): 2996 (C–H stretch), 1669 (C=O carbonyl stretch), 1596, 1570, 1557 (C–C aromatic stretch); Elemental analysis: Calculated for $C_{17}H_{16}N_2O$ (MW 264.33): 77.25% C; 6.10% H; 10.60% N. Found: 77.11% C; 6.24% H; 10.65% N.

**3-(3-Chlorobenzyl)-2-methylquinazolin-4(3*H*)-one (6).** Yellow solid; Yield 49%; m.p. 171–172 °C; $^1H$ NMR (600 MHz, DMSO-$d_6$): δ 8.13 – 8.09 (m, 1H), 7.81 – 7.76 (m, 1H), 7.61 – 7.56 (m, 1H), 7.51 – 7.45 (m, 1H), 7.36 – 7.29 (m, 2H), 7.28 (d, *J* = 2.0 Hz, 1H), 7.13 – 7.08 (m, 1H), 5.33 (s, 2H, $CH_2$), 2.45 (s, 3H, $CH_3$); $^{13}C$ NMR (151 MHz, DMSO-$d_6$): δ 162.06, 155.49, 147.65, 139.67, 135.13, 133.98, 131.26, 127.93, 127.21, 127.05, 126.96, 126.93, 125.50, 120.39, 46.56, 23.51; IR (ATR-Ge, $cm^{-1}$): 3010 (C–H stretch), 1672 (C=O carbonyl stretch), 1638, 1594, 1571 (C–C aromatic stretch); Elemental analysis: Calculated for $C_{16}H_{13}ClN_2O$ (MW 284.74): 67.49% C; 4.60% H; 9.84% N. Found: 67.39% C; 4.55% H; 9.87% N.

**2-Methyl-3-(4-(trifluoromethyl)benzyl)quinazolin-4(3*H*)-one (7).** White solid; Yield 60%; m.p. 174–176 °C; $^1H$ NMR (600 MHz, DMSO-$d_6$): δ 8.20 – 8.16 (m, 2H), 7.89 – 7.84 (m, 2H), 7.57 – 7.52 (m, 2H), 7.17 – 7.11 (m, 2H), 4.28 (s, 2H, $CH_2$), 2.08 (s, 3H, $CH_3$); $^{13}C$ NMR (151 MHz, DMSO-$d_6$): δ 162.06, 155.49, 147.65, 139.67, 135.13, 133.98, 131.26, 127.93, 127.21, 127.05, 126.96, 126.93, 125.50, 120.39, 46.56, 23.51; IR (ATR-Ge, $cm^{-1}$): 3066 (C–H stretch), 1656 (C=O carbonyl stretch), 1592, 1569, 1516 (C–C aromatic stretch).

**2-Methyl-3-(naphthalen-2-ylmethyl)quinazolin-4(3*H*)-one (8).** Yellow solid; Yield 88%; m.p. 178–179 °C; $^1H$ NMR (600 MHz, $CDCl_3$): δ 8.32 – 8.28 (m, 1H), 8.06 – 8.00 (m, 1H), 7.94 – 7.87 (m, 1H), 7.81 – 7.74 (m, 2H), 7.71 – 7.67 (m, 1H), 7.64 – 7.44 (m, 2H), 7.43 – 7.28 (m, 2H), 6.84 – 6.80 (m, 1H), 5.82 (s, 2H, $CH_2$), 2.47 (s, 3H, $CH_3$); $^{13}C$ NMR (151 MHz, $CDCl_3$): δ 169.13, 168.82, 162.37, 154.98, 147.55, 139.76, 134.65, 133.87, 132.75, 130.74, 129.03, 128.18, 126.96, 125.62, 123.34, 122.72, 121.57, 120.38, 44.86, 23.13; IR (ATR-Ge, $cm^{-1}$): 3056 (C–H stretch), 1605 (C=O carbonyl stretch), 1621, 1594, 1570 (C–C aromatic stretch); Elemental analysis: Calculated for $C_{20}H_{16}N_2O$ (MW 300.36): 79.98% C; 5.37% H; 9.33% N. Found: 79.88% C; 5.47% H; 9.55% N.

**3-(3-Fluoro-5-(trifluoromethyl)benzyl)-2-methylquinazolin-4(3*H*)-one (9).** Light beige solid; Yield 76%; m.p. 176–177 °C; $^1H$ NMR (600 MHz, $CDCl_3$): δ 8.32 – 8.28 (m, 1H), 7.94 – 7.87 (m, 1H), 7.81 – 7.74 (m, 1H), 7.71 – 7.67 (m, 1H), 7.64 – 7.44 (m, 1H), 7.43 – 7.28 (m, 1H), 6.84 – 6.80 (m, 1H), 5.82 (s, 2H, $CH_2$), 2.47 (s, 3H, $CH_3$); $^{13}C$ NMR (151 MHz, DMSO-$d_6$): δ 162.06, 155.49, 147.65, 139.67, 135.13, 133.98, 131.26, 127.93, 127.21, 127.05, 126.96, 126.93, 125.50, 120.39, 46.56, 23.51; IR (ATR-Ge, $cm^{-1}$): 3056 (C–H stretch), 1605 (C=O carbonyl stretch), 1621, 1594, 1570 (C–C aromatic stretch).

**3-(2,4-Dichlorobenzyl)-2-methylquinazolin-4(3*H*)-one (10).** White solid; Yield 46%; m.p. 172–173 °C; $^1H$ NMR (600 MHz, $CDCl_3$): δ 8.32 – 8.25 (m, 1H), 7.80 – 7.74 (m, 1H), 7.66 (d, *J* = 8.2 Hz, 1H), 7.51 – 7.43 (m, 2H), 7.17 – 7.12 (m, 1H), 6.79 – 6.74 (m, 1H), 5.42 (s, 2H, $CH_2$), 2.49 (s, 3H, $CH_3$); $^{13}C$ NMR (151 MHz, $CDCl_3$): δ 162.33, 154.21, 147.43, 134.80, 134.17,

133.24, 131.97, 129.74, 129.63, 127.87, 127.47, 127.21, 127.01, 120.23, 44.56, 23.14; IR (ATR-Ge, cm$^{-1}$): 3069 (C–H stretch), 1678 (C=O carbonyl stretch), 1595, 1566 (C–C aromatic stretch); Elemental analysis: Calculated for $C_{16}H_{12}Cl_2N_2O$ (MW 319.19): 60.21% C; 3.79% H; 8.78% N. Found: 60.30% C; 3.70% H; 8.54% N.

**(3,4-Dichlorobenzyl)-2-methylquinazolin-4(3*H*)-one (11).** White solid; Yield 56%; m.p. 174–175 °C; $^1$H NMR (600 MHz, $CDCl_3$): δ 8.25 – 8.20 (m, 1H), 7.73 – 7.67 (m, 1H), 7.61 – 7.56 (m, 1H), 7.47 – 7.39 (m, 1H), 7.33 (d, *J* = 8.3 Hz, 1H), 7.02 – 6.98 (m, 2H), 5.26 (s, 2H, $CH_2$), 2.10 (s, 3H, $CH_3$); $^{13}$C NMR (151 MHz, $CDCl_3$): δ 162.29, 154.01, 147.34, 136.30, 134.73, 133.24, 132.04, 131.00, 128.71, 127.13, 126.95, 126.85, 126.10, 120.28, 46.32, 23.47; IR (ATR-Ge, cm$^{-1}$): 3069 (C–H stretch), 1678 (C=O carbonyl stretch), 1595, 1566 (C–C aromatic stretch); Elemental analysis: Calculated for $C_{16}H_{12}Cl_2N_2O$ (MW 319.19): 60.21% C; 3.79% H; 8.78% N. Found: 60.29% C; 3.71% H; 8.48% N.

**3-((7-Chloro-2-methyl-4-oxoquinazolin-3(4*H*)-yl)methyl)benzoic acid (12).** White solid; Yield 18 %; m.p. 235.5–236.4 °C; LRMS (APCI) m/z: [M-H]$^-$ = 327.0, [M+H]$^+$ = 329.5 (exact mass = 328.06); $^1$H NMR (600 MHz, DMSO-$d_6$): δ 12.98 (s, 1H), 8.11 (d, 1H), 7.86 – 7.79 (m, 1H), 7.77 – 7.69 (m, 1H), 7.64 (d, 1H), 7.51 (dd, 1H), 7.45 – 7.43 (m, 2H), 5.39 (s, 2H), 2.46 (s, 3H); $^{13}$C NMR (151 MHz, DMSO-$d_6$): δ 167.52, 161.49, 157.31, 148.70, 139.79, 137.35, 131.80, 131.46, 129.72, 129.10, 128.90, 127.64, 127.41, 126.37, 119.17, 46.86, 23.61; Calculated for $C_{17}H_{13}ClN_2O_3$ (MW 328.75) 62.11 % C; 3.99 % H; 8.52 % N. Found: 61.92 % C; 4.11 % H; 8.35 % N.

**7-Chloro-3-(3-hydroxybenzyl)-2-methylquinazolin-4(3*H*)-one (13).** White solid; Yield 29 %; m.p. 247.1–247.7 °C; LRMS (APCI) m/z: [M-H]$^-$ = 298.4, [M+H]$^+$ = 301.3 (exact mass = 300.07); $^1$H NMR (600 MHz, DMSO-$d_6$): δ 9.38 (s, 1H), 8.11 (d, 1H), 7.64 (d, 1H), 7.51 (dd, 1H), 7.10 (t, 1H), 6.65 – 6.56 (m, 2H), 6.50 (t, 1H), 5.25 (s, 2H), 2.45 (s, 3H); $^{13}$C NMR (151 MHz, DMSO-$d_6$): δ 161.40, 158.29, 157.47, 148.71, 139.70, 138.13, 130.41, 129.11, 127.33, 126.33, 119.19, 117.38, 114.90, 113.30, 46.81, 23.49; Calculated for $C_{16}H_{13}ClN_2O_2$ (MW 300.74) 63.90 % C; 4.36 % H; 9.31 % N. Found: 64.03 % C; 4.41 % H; 10.12 % N.

**7-Chloro-3-(4-hydroxybenzyl)-2-methylquinazolin-4(3*H*)-one (14).** Dark beige solid; Yield 52%; m.p. 178–179 °C; $^1$H NMR (600 MHz, DMSO-$d_6$): δ 9.37 (s, 1H), 8.09 (d, *J* = 8.5 Hz, 1H), 7.61 (d, *J* = 2.0 Hz, 1H), 7.51 – 7.46 (m, 1H), 7.03 – 6.98 (m, 2H), 6.71 – 6.66 (m, 2H), 5.20 (s, 2H, $CH_2$), 2.06 (s, 3H, $CH_3$); $^{13}$C NMR (151 MHz, DMSO-$d_6$): δ 161.50, 157.47, 157.25, 148.70, 140.92, 139.59, 129.26, 129.09, 128.50, 127.22, 126.84, 126.26, 116.06, 115.62, 46.56, 23.56; IR (ATR-Ge, cm$^{-1}$): 3293 (N–H stretch), 2910 (C–H stretch), 1677 (C=O carbonyl stretch), 1621, 1590 (C–C aromatic stretch); Elemental analysis: Calculated for $C_{16}H_{13}ClN_2O_2$ (MW 300.74): 63.90% C; 4.36% H; 9.31% N. Found: 63.82% C; 4.44% H; 9.45% N.

**7-Chloro-3-(2,4-dimethoxybenzyl)-2-methylquinazolin-4(3*H*)-one (15).** Yellow solid; Yield 16%; m.p. 175–177 °C; $^1$H NMR (600 MHz, DMSO-$d_6$): δ 8.07 (d, *J* = 8.6 Hz, 1H), 7.64 (d, *J* = 2.1 Hz, 1H), 7.52 – 7.47 (m, 1H), 6.63 (d, *J* = 8.4 Hz, 1H), 6.58 (d, *J* = 2.4 Hz, 1H), 6.41 – 6.36 (m, 1H), 5.13 (s, 2H, $CH_2$), 3.80 (s, 3H, $OCH_3$), 3.69 (s, 3H, $OCH_3$), 2.43 (s, 3H, $CH_3$); $^{13}$C NMR (151 MHz, DMSO-$d_6$): δ 161.38, 160.47, 157.88, 157.58, 148.75, 139.59, 129.01, 127.23, 126.28, 119.28, 116.17, 105.46, 99.09, 56.13, 55.76, 42.56, 23.18; IR (ATR-Ge, cm$^{-1}$): 3293 (N–H stretch), 2910 (C–H stretch), 1677 (C=O carbonyl stretch), 1621, 1590 (C–C aromatic stretch); Elemental analysis: Calculated for $C_{18}H_{17}ClN_2O_3$ (MW 344.79): 62.70% C; 4.97% H; 8.12% N. Found: 62.75% C; 4.92% H; 8.13% N.

**7-Chloro-3-(3-methoxybenzyl)-2-methylquinazolin-4(3*H*)-one (16).** Yellow solid; Yield 26%; m.p. 170–171 °C; $^1$H NMR (600 MHz, DMSO-$d_6$): δ 8.10 (d, *J* = 8.6 Hz, 1H), 7.78 (d, *J* = 8.4 Hz, 1H), 7.64 (d, *J* = 2.1 Hz, 1H), 7.53 – 7.48 (m, 1H), 7.24

– 7.17 (m, 3H), 5.30 (s, 2H, $CH_2$), 3.70 (s, 3H, $OCH_3$), 2.06 (s, 3H, $CH_3$); $^{13}C$ NMR (151 MHz, $CDCl_3$): δ 161.86, 160.21, 156.12, 148.39, 138.94, 137.26, 130.19, 128.70, 127.27, 126.45, 118.71, 112.84, 112.68, 55.33, 47.19, 23.53. IR (ATR-Ge, $cm^{-1}$): 3361 (N–H stretch), 2922 (C–H stretch), 1674 (C=O carbonyl stretch), 1600, 1577 (C–C aromatic stretch); Elemental analysis: Calculated for $C_{17}H_{15}ClN_2O_2$ (MW 314.77): 64.87% C; 4.80% H; 8.90% N. Found: 64.78% C; 4.89% H; 8.89% N.

**7-Chloro-3-(4-methoxybenzyl)-2-methylquinazolin-4(3*H*)-one (17).** Light beige solid; Yield 26%; m.p. 172–173 °C; $^1H$ NMR (600 MHz, $CDCl_3$): δ 8.20 (d, *J* = 8.5 Hz, 1H), 7.60 (d, *J* = 2.0 Hz, 1H), 7.41 – 7.38 (m, 1H), 7.16 – 7.10 (m, 2H), 6.91 – 6.81 (m, 2H), 5.29 (s, 2H, $CH_2$), 3.76 (s, 3H, $OCH_3$), 2.54 (s, 3H, $CH_3$); $^{13}C$ NMR (151 MHz, $CDCl_3$): δ 161.96, 159.31, 156.12, 148.40, 140.62, 129.39, 128.68, 128.18, 127.73, 127.22, 126.43, 114.47, 55.39, 46.88, 23.60; IR (ATR-Ge, $cm^{-1}$): 3325 (N–H stretch), 2960 (C–H stretch), 1674 (C=O carbonyl stretch), 1603, 1585, 1564 (C–C aromatic stretch); Elemental analysis: Calculated for $C_{17}H_{15}ClN_2O_2$ (MW 314.77) 64.87% C; 4.80% H; 8.90% N. Found: 64.76% C; 4.91% H; 8.80% N.

**7-Chloro-3-(2-fluorobenzyl)-2-methylquinazolin-4(3*H*)-one (18).** Light beige solid; Yield 24%; m.p. 168–169 °C; $^1H$ NMR (600 MHz, DMSO-$d_6$): δ 8.07 (d, *J* = 8.5 Hz, 1H), 7.65 (d, *J* = 2.0 Hz, 1H), 7.52 – 7.47 (m, 1H), 7.34 – 7.28 (m, 1H), 7.25 – 7.19 (m, 1H), 7.10 (t, *J* = 7.5 Hz, 1H), 7.02 – 6.96 (m, 1H), 5.33 (s, 2H , $CH_2$), 2.48 (s, 3H , $CH_3$); $^{13}C$ NMR (151 MHz, DMSO-$d_6$): δ 161.33, 159.45, 157.25, 148.71, 139.75, 130.00 (d, *J* = 32 Hz), 129.02, 128.18, 127.37, 126.36 (d, *J* = 272.4 Hz), 125.42, 123.56, 119.19 (d *J* = 6 Hz), 116.14 (d, *J* = 4 Hz), 41.83, 23.33; IR (ATR-Ge, $cm^{-1}$): 3298 (N–H stretch), 2990 (C–H stretch), 1680 (C=O carbonyl stretch), 1601, 1564 (C–C aromatic stretch).

**7-Chloro-3-(4-fluorobenzyl)-2-methylquinazolin-4(3*H*)-one (19).** Yellow solid; Yield 22%; m.p. 167–168 °C; $^1H$ NMR (600 MHz, DMSO-$d_6$): δ 8.09 (d, *J* = 8.5 Hz, 1H), 7.62 (d, *J* = 2.1 Hz, 1H), 7.51 – 7.47 (m, 1H), 7.27 – 7.21 (m, 2H), 7.21 – 7.09 (m, 2H), 5.30 (s, 2H. $CH_2$), 2.45 (s, 3H, $CH_3$); $^{13}C$ NMR (151 MHz, DMSO-$d_6$): δ 162.75, 161.49, 157.29, 148.70, 139.69, 132.97 (d, *J* = 33 Hz), 132.95, 129.19, 129.07, 127.30, 126.31 (d *J* = 272.2 Hz), 119.27, 116.19 (d, *J* = 5 Hz), 116.05 (d, *J* = 4 Hz), 46.46, 23.57; IR (ATR-Ge, $cm^{-1}$): 3283 (N–H stretch), 2956 (C–H stretch), 1672 (C=O carbonyl stretch), 1601, 1592 (C–C aromatic stretch).

**7-Chloro-2-methyl-3-(2-methylbenzyl)quinazolin-4(3*H*)-one (20).** Yellow solid; Yield 43%; m.p. 168–170 °C; $^1H$ NMR (600 MHz, $CDCl_3$): δ 8.21 (d, *J* = 8.3 Hz, 1H), 7.65 (d, *J* = 2.0 Hz, 1H), 7.44 – 7.39 (m, 1H), 7.25 (s, 1H), 7.24 – 7.12 (m, 1H), 7.11 – 7.05 (m, 1H), 6.64 (d, *J* = 7.4 Hz, 1H), 5.31 (s, 2H , $CH_2$), 2.47 (s, 3H , $CH_3$), 2.41 (s, 3H , $CH_3$); $^{13}C$ NMR (151 MHz, DMSO-$d_6$): δ 161.42, 158.31, 157.49, 148.73, 139.71, 138.15, 130.43, 129.12, 127.34, 126.34, 119.20, 117.39, 114.91, 113.31, 46.81, 40.61, 23.49; IR (ATR-Ge, $cm^{-1}$): 3375 (N–H stretch), 2943 (C–H stretch), 1682 (C=O carbonyl stretch), 1629, 1593, 1562 (C–C aromatic stretch); Elemental analysis: Calculated for $C_{17}H_{15}ClN_2O$ (MW 298.77) 68.34% C; 5.06% H; 9.38% N. Found: 68.25% C; 5.15% H; 9.37% N.

**7-Chloro-3-(3-chlorobenzyl)-2-methylquinazolin-4(3*H*)-one (21).** Light yellow solid; Yield 25%; m.p. 165–166 °C; $^1H$ NMR (600 MHz, $CDCl_3$): δ 8.21 (d, *J* = 8.5 Hz, 1H), 7.62 (d, *J* = 2.0 Hz, 1H), 7.44 – 7.38 (m, 1H), 7.30 – 7.22 (m, 2H), 7.19 – 7.13 (m, 1H), 7.09 – 7.03 (m, 1H), 5.33 (s, 2H , $CH_2$), 2.52 (s, 3H, $CH_3$); $^{13}C$ NMR (151 MHz, $CDCl_3$): δ 161.82, 155.69, 148.37, 140.88, 137.75, 135.14, 130.42, 128.71, 128.25, 127.45, 126.79, 126.58, 124.76, 118.84, 46.82, 23.57; IR (ATR-Ge, $cm^{-1}$): 3388 (N–H stretch), 2967 (C–H stretch), 1673 (C=O carbonyl stretch), 1621, 1592 (C–C aromatic stretch); Elemental analysis: Calculated for $C_{16}H_{12}Cl_2N_2O$ (MW 319.19) 60.21% C; 3.79% H; 8.78% N. Found: 60.32% C; 3.68% H; 8.67% N.

**7-Chloro-2-methyl-3-(4-(trifluoromethyl)benzyl)quinazolin-4(3*H*)-one (22).** Yellow solid; Yield 22%; m.p. 171–172 °C; $^{1}$H NMR (600 MHz, $CDCl_3$): δ 8.20 (d, *J* = 8.5 Hz, 1H), 7.65 – 7.56 (m, 2H), 7.46 – 7.37 (m, 2H), 7.32 – 7.27 (m, 2H), 5.40 (s, 2H , $CH_2$), 2.52 (s, 3H , $CH_3$); $^{13}$C NMR (151 MHz, $CDCl_3$): N/A; IR (ATR-Ge, cm$^{-1}$): 3366 (N–H stretch), 2940 (C–H stretch), 1681 (C=O carbonyl stretch), 1645, 1608, 1588 (C–C aromatic stretch).

**7-Chloro-2-methyl-3-(naphthalen-2-ylmethyl)quinazolin-4(3*H*)-one (23).** Light yellow solid; Yield 34%; m.p. 175–176 °C; $^{1}$H NMR (600 MHz, $CDCl_3$): δ 8.23 (d, *J* = 8.6 Hz, 1H), 8.03 (d, *J* = 8.3 Hz, 1H), 7.91 (dd, *J* = 8.3, 1.4 Hz, 1H), 7.85 – 7.75 (m, 1H), 7.69 (d, *J* = 2.0 Hz, 1H), 7.64 – 7.59 (m, 1H), 7.59 – 7.53 (m, 1H), 7.48 – 7.38 (m, 1H), 7.36 – 7.30 (m, 1H), 6.85 – 6.79 (m, 1H), 5.83 (s, 2H , $CH_2$), 2.48 (s, 3H , $CH_3$); $^{13}$C NMR (151 MHz, $CDCl_3$): δ 161.77, 156.40, 148.50, 140.83, 133.89, 130.47, 129.22, 128.79, 128.30, 127.41, 126.78, 126.55, 126.29, 125.61, 125.52, 122.15, 121.45, 118.84, 44.93, 23.21; IR (ATR-Ge, cm$^{-1}$): 3386 (N–H stretch), 2942 (C–H stretch), 1683 (C=O carbonyl stretch), 1589, 1561 (C–C aromatic stretch); Elemental analysis: Calculated for $C_{20}H_{15}ClN_2O$ (MW 334.80) 71.75% C; 4.52% H; 8.37% N. Found: 71.64% C; 4.61% H; 8.38% N.

**7-Chloro-3-(3,4-dichlorobenzyl)-2-methylquinazolin-4(3*H*)-one (24).** Light beige solid; Yield 30%; m.p. 170–172 °C; $^{1}$H NMR (600 MHz, $CDCl_3$): δ 8.64 (d, *J* = 2.1 Hz, 1H), 8.18 (d, *J* = 8.4 Hz, 1H), 7.62 (d, *J* = 2.0 Hz, 1H), 7.43 – 7.37 (m, 2H), 7.18 – 7.13 (m, 1H), 5.27 (s, 2H, $CH_2$), 2.17 (s, 3H, $CH_3$); $^{13}$C NMR (151 MHz, DMSO-*d*$_6$): δ 169.19, 168.06, 161.53, 157.16, 148.72, 138.03, 137.00, 131.45, 130.40, 129.34, 126.34, 122.92, 120.40, 119.29, 46.30, 23.63; IR (ATR-Ge, cm$^{-1}$): 3361 (N–H stretch), 2924 (C–H stretch), 1677 (C=O carbonyl stretch), 1599, 1581 (C–C aromatic stretch); Elemental analysis: Calculated for $C_{16}H_{11}Cl_3N_2O$ (MW 353.63) : 54.34% C; 3.14% H; 7.92% N. Found: 54.23% C; 3.25% H; 7.71% N.

**(*E*)-7-Chloro-3-((4-hydroxy-3-nitrobenzylidene)amino)-2-methylquinazolin-4(3*H*)-one (25).** Yellow solid; Yield 13 %; m.p. 253.4–254.2 °C; LRMS (APCI) m/z: $[M-H]^-$ = 356.6, $[M+H]^+$ = 359.2 (exact mass = 358.05); $^{1}$H NMR (600 MHz, DMSO-*d*$_6$): δ 8.85 (s, 1H, –CH=), 8.38 (d, *J* = 2.2 Hz, 1H), 8.12 (d, *J* = 8.6 Hz, 1H), 8.06 (dd, *J* = 8.8, 2.2 Hz, 1H), 7.71 (d, *J* = 2.1 Hz, 1H), 7.54 (dd, *J* = 8.5, 2.1 Hz, 1H), 7.20 – 7.16 (d, 2H), 2.51 (s, 3H, $CH_3$); $^{13}$C NMR: N/A; Elemental analysis: Calculated for $C_{16}H_{11}ClN_4O_4$ (MW 358.74) 53.57 % C; 3.09 % H; 15.62 % N. Found: 54.11 % C; 3.23 % H; 16.23 % N.

**(*E*)-7-Chloro-3-((2-nitrobenzylidene)amino)-2-methylquinazolin-4(3*H*)-one (26).** Yellow solid; Yield 14 %; m.p. 204.3–204.9 °C; LRMS (APCI) m/z: $[M-H]^-$ = 340.7, $[M+H]^+$ = 343.3 (exact mass = 342.05); $^{1}$H NMR (600 MHz, $CDCl_3$): δ 9.62 (s, 1H, –CH=), 8.22 – 8.14 (m, 3H), 7.82 – 7.77 (m, 1H), 7.75 – 7.69 (m, 1H), 7.66 (d, 1H), 7.41 (dd, 1H), 2.65 (s, 3H, $CH_3$); $^{13}$C NMR (151 MHz, $CDCl_3$): δ 163.14, 158.22, 155.31, 148.84, 147.41, 140.88, 133.99, 132.40, 129.99, 128.91, 128.22, 127.35, 126.75, 125.01, 120.02, 22.99; Elemental analysis: Calculated for $C_{16}H_{11}ClN_4O_3$ (MW 342.74) 56.07 % C; 3.24 % H; 16.35 % N. Found: 56.34 % C; 3.56 % H; 16.08 % N.

**(*E*)-7-Chloro-3-((3-nitrobenzylidene)amino)-2-methylquinazolin-4(3*H*)-one (27).** Light yellow solid; Yield 22 %; m.p. 198.5–199.2 °C; LRMS (APCI) m/z: $[M-H]^-$ = 340.4, $[M+H]^+$ = 343.3 (exact mass = 342.05); $^{1}$H NMR (600 MHz, $CDCl_3$): δ 9.37 (s, 1H, –CH=), 8.73 (t, 1H), 8.42 – 8.37 (m, 1H), 8.24 – 8.16 (m, 2H), 7.71 (t, 1H), 7.67 (d, 1H), 7.43 (dd, 1H), 2.71 (s, 3H, $CH_3$); $^{13}$C NMR (151 MHz, $CDCl_3$): δ 162.28, 158.58, 155.54, 148.85, 147.28, 141.01, 134.86, 134.27, 130.22, 128.86, 127.44, 126.80, 126.64, 123.24, 119.99, 23.15; Elemental analysis: Calculated for $C_{16}H_{11}ClN_4O_3$ (MW 342.74) 56.07 % C; 3.24 % H; 16.35 % N. Found: 56.61 % C; 3.22 % H; 15.86 % N.

**(*E*)-7-Chloro-3-((4-nitrobenzylidene)amino)-2-methylquinazolin-4(3*H*)-one (28).** Yellow solid; Yield 16 %; m.p. 228.4–228.7 °C; LRMS (APCI) m/z: $[M-H]^-$ = 340.6, $[M+H]^+$ = 343.7 (exact mass = 342.05); $^{1}$H NMR (600 MHz, $CDCl_3$): δ 9.45 (s,

1H, –CH=), 8.36 – 8.33 (m, 2H), 8.20 (d, 1H), 8.08 – 8.00 (m, 2H), 7.67 (d, 1H), 7.43 (dd, 1H), 2.70 (s, 3H, $CH_3$); $^{13}C$ NMR (151 MHz, $CDCl_3$): N/A Elemental analysis: Calculated for $C_{16}H_{11}ClN_4O_3$ (MW 342.74) 56.07 % C; 3.24 % H; 16.35 % N. Found: 56.26 % C; 3.34 % H; 15.98 % N.

**(*E*)-7-Chloro-3-((pyridin-2-ylmethylene)amino)-2-methylquinazolin-4(3*H*)-one (29).** Beige solid; Yield 47 %; m.p. 198.2–198.6 °C; LRMS (APCI) m/z: $[M-H]^-$ = 297.1, $[M+H]^+$ = 300.2 (exact mass = 298.06); $^1H$ NMR (600 MHz, $CDCl_3$): δ 9.22 (s, 1H, –CH=), 9.00 (d, 1H), 8.77 – 8.73 (m, 1H), 8.25 – 8.20 (m, 1H), 8.17 (d, 1H), 7.64 (d, 1H), 7.43 (dd, 1H), 7.39 (dd, 1H), 2.65 (s, 3H, $CH_3$); $^{13}C$ NMR (151 MHz, $CDCl_3$): δ 162.78, 158.46, 155.49, 153.15, 150.65, 147.33, 140.86, 134.96, 128.96, 128.80, 127.33, 126.74, 124.02, 120.01, 23.05; Elemental analysis: Calculated for $C_{15}H_{11}ClN_4O$ (MW 298.73) 60.31 % C; 3.71 % H; 18.76 % N. Found: 59.88 % C; 3.81 % H; 18.46 % N.

**(*E*)-7-Chloro-3-((pyridin-3-ylmethylene)amino)-2-methylquinazolin-4(3*H*)-one (30).** Beige solid; Yield 42 %; m.p. 199.3–199.8 °C; LRMS (APCI) m/z: $[M-H]^-$ = 297.0, $[M+H]^+$ = 299.9 (exact mass = 298.06); $^1H$ NMR (600 MHz, $CDCl_3$): δ 9.23 (s, 1H, –CH=), 9.01 (d, 1H), 8.81 – 8.74 (m, 1H), 8.26 – 8.21 (m, 1H), 8.18 (d, 1H), 7.65 (d, 1H), 7.44 (dd, 1H), 7.40 (dd, 1H), 2.66 (s, 3H, $CH_3$); $^{13}C$ NMR (151 MHz, $CDCl_3$): δ 162.78, 158.46, 155.49, 153.15, 150.65, 147.33, 140.86, 134.96, 128.96, 128.80, 127.33, 126.74, 124.02, 120.01, 23.05; Elemental analysis: Calculated for $C_{15}H_{11}ClN_4O$ (MW 298.73) 60.31 % C; 3.71 % H; 18.76 % N. Found: 60.57 % C; 3.84 % H; 18.49 % N.

**(*E*)-7-Chloro-3-((pyridin-4-ylmethylene)amino)-2-methylquinazolin-4(3*H*)-one (31).** Beige solid; Yield 58 %; m.p. 203.1–203.7 °C; LRMS (APCI) m/z: $[M-H]^-$ = 296.8, $[M+H]^+$ = 299.6 (exact mass = 298.06); $^1H$ NMR (600 MHz, $CDCl_3$): δ 9.33 (s, 1H, –CH=), 8.81 – 8.77 (m, 2H), 8.19 (d, 1H), 7.74 – 7.69 (m, 2H), 7.65 (d, 1H), 7.41 (dd, 1H), 2.69 (s, 3H, $CH_3$); $^{13}C$ NMR (151 MHz, $CDCl_3$): δ 162.10, 158.66, 155.65, 150.89, 147.22, 141.04, 140.26, 128.84, 127.46, 126.81, 122.04, 120.04, 23.13; Elemental analysis: Calculated for $C_{15}H_{11}ClN_4O$ (MW 298.73) 60.31 % C; 3.71 % H; 18.76 % N. Found: 60.46 % C; 3.80 % H; 17.87 % N.

**(*E*)-7-Chloro-3-((2,3-dihydroxybenzylidene)amino)-2-methylquinazolin-4(3*H*)-one (32).** White solid; Yield 36 %; m.p. 261.7–262.3 °C; LRMS (APCI) m/z: $[M-H]^-$ = 327.1, $[M+H]^+$ = 330.4 (exact mass = 329.06); $^1H$ NMR (600 MHz, $CDCl_3$): δ 10.86 (s, 1H, –CH=), 8.96 (d, 1H), 8.21 (d, 1H), 7.69 (s, 1H, OH), 7.44 (d, 1H), 7.15 (d, 1H), 6.95 (d, 2H), 5.70 (s, 1H, OH), 2.65 (s, 3H, $CH_3$); $^{13}C$ NMR: N/A; Elemental analysis: Calculated for $C_{16}H_{12}ClN_3O_3$ (MW 329.74) 58.28 % C; 3.67 % H; 12.74 % N. Found: 58.56 % C; 3.74 % H; 12.47 % N.

**(*E*)-7-Chloro-3-((2,4-dihydroxybenzylidene)amino)-2-methylquinazolin-4(3*H*)-one (33).** White solid; Yield 21 %; m.p. 272.3–273.0 °C; LRMS (APCI) m/z: $[M-H]^-$ = 327.0, $[M+H]^+$ = 330.6 (exact mass = 329.06); $^1H$ NMR (600 MHz, $CDCl_3$): δ 9.21 (s, 1H, –CH=), 8.18 (dd, 1H), 7.65 (d, 1H), 7.62 – 7.58 (m, 1H), 7.56 – 7.52 (m, 1H), 7.39 (dd, 1H), 7.17 (dd, 1H), 2.65 (s, 3H, $CH_3$); $^{13}C$ NMR (151 MHz, $CDCl_3$): δ 159.66, 158.50, 155.62, 147.33, 140.61, 137.47, 134.54, 131.88, 128.72, 128.12, 127.14, 126.60, 119.95, 22.94.; Calculated for $C_{16}H_{12}ClN_3O_3$ (MW 329.74) 58.28 % C; 3.67 % H; 12.74 % N. Found: 58.63 % C; 3.78 % H; 12.37 % N.

**(*E*)-7-Chloro-3-((4-hydroxy-3-methoxybenzylidene)amino)-2-methylquinazolin-4(3*H*)-one (34).** Beige solid; Yield 64 %; m.p. 243.6–244.5 °C; LRMS (APCI) m/z: $[M-H]^-$ = 341.8, $[M+H]^+$ = 344.4 (exact mass = 343.07); $^1H$ NMR (600 MHz, DMSO-$d_6$): δ 9.99 (s, 1H, –CH=), 8.66 (s, 1H, OH), 8.09 (d, 1H), 7.67 (d, 1H), 7.53 – 7.48 (m, 2H), 7.31 (dd, 1H), 6.91 (d, 1H), 3.82 (s, 3H, $OCH_3$), 2.69 (s, 3H, $CH_3$) ; $^{13}C$ NMR (151 MHz, DMSO-$d_6$): δ 170.26, 157.55, 155.65, 152.11, 148.70, 147.96, 139.35,

129.18, 127.05, 126.41, 125.20, 123.98, 120.40, 116.16, 111.14, 56.20, 22.81; Calculated for $C_{17}H_{14}ClN_3O_3$ (MW 343.77) 59.40 % C; 4.11 % H; 12.22 % N. Found: 59.57 % C; 4.17 % H; 12.65 % N.

**(*E*)-7-Chloro-3-((2-hydroxybenzylidene)amino)-2-methylquinazolin-4(3*H*)-one (35).** Beige solid; Yield 32 %; m.p. 228.2–228.6 °C; LRMS (APCI) m/z: $[M-H]^-$ = 311.5, $[M+H]^+$ = 314.3 (exact mass = 313.06); $^1H$ NMR (600 MHz, $CDCl_3$): δ 10.62 (s, 1H, –CH=), 9.00 (s, 1H, OH), 8.20 (d, 1H), 7.68 (d, 1H), 7.49 – 7.45 (m, 1H), 7.42 (dd, 1H), 7.37 (dd, 1H), 7.07 (dd, 1H), 7.03 – 6.97 (m, 1H), 2.64 (s, 3H, $CH_3$); $^{13}C$ NMR (151 MHz, $CDCl_3$): δ 170.92, 160.28, 157.98, 153.99, 147.42, 140.95, 134.75, 133.55, 128.75, 127.40, 126.85, 120.05, 119.86, 117.68, 116.39, 22.88; Elemental analysis: Calculated for $C_{16}H_{12}ClN_3O_2$ (MW 313.74) 61.25 % C; 3.86 % H; 13.39 % N. Found: 60.96 % C; 3.74 % H; 12.98 % N.

**(*E*)-7-Chloro-3-((3-hydroxybenzylidene)amino)-2-methylquinazolin-4(3*H*)-one (36).** White solid; Yield 53 %; m.p. 243.8–244.6 °C; LRMS (APCI) m/z: $[M-H]^-$ = 311.8, $[M+H]^+$ = 314.5 (exact mass = 313.06); $^1H$ NMR (600 MHz, DMSO-$d_6$): δ 9.63 (s, 1H, –CH=), 8.84 (s, 1H, OH), 8.11 (d, 1H), 7.65 (d, 1H), 7.50 (dd, 1H), 7.37 – 7.31 (m, 2H), 7.33 – 7.28 (m, 1H), 7.03 – 6.98 (m, 1H), 2.49 (s, 3H, $CH_3$); $^{13}C$ NMR (151 MHz, DMSO-$d_6$): δ 170.02, 158.41, 157.53, 155.55, 147.96, 139.51, 134.11, 130.67, 129.21, 127.08, 126.45, 120.91, 120.64, 120.51, 114.96, 22.69; Elemental analysis: Calculated for $C_{16}H_{12}ClN_3O_2$ (MW 313.74) 61.25 % C; 3.86 % H; 3.39 % N. Found: 61.36 % C; 3.84 % H; 13.17 % N.

**(*E*)-7-Chloro-3-((4-hydroxybenzylidene)amino)-2-methylquinazolin-4(3*H*)-one (37).** White solid; Yield 44 %; m.p. 281.8–282.6 °C; LRMS (APCI) m/z: $[M-H]^-$ = 311.7, $[M+H]^+$ = 314.3 (exact mass = 313.06); $^1H$ NMR (600 MHz, DMSO-$d_6$): δ 10.39 (s, 1H, –CH=), 8.71 (s, 1H, OH), 8.11 (d, 1H), 7.82 – 7.76 (m, 2H), 7.69 (d, 1H), 7.53 (dd, 1H), 6.96 – 6.91 (m, 2H), 2.48 (s, 3H, $CH_3$); $^{13}C$ NMR (151 MHz, DMSO-$d_6$): δ 170.06, 162.45, 157.55, 155.63, 147.94, 139.32, 131.59, 129.15, 127.02, 126.39, 123.66, 120.40, 116.55, 22.78; Elemental analysis: Calculated for $C_{16}H_{12}ClN_3O_2$ (MW 313.74) 61.25 % C; 3.86 % H; 13.39 % N. Found: 61.52 % C; 3.93 % H; 13.06 % N.

**(*E*)-7-Chloro-3-((2-methoxybenzylidene)amino)-2-methylquinazolin-4(3*H*)-one (38).** White solid; Yield 68 %; m.p. 195.4–196.2 °C; LRMS (APCI) m/z: $[M-H]^-$ = 325.7, $[M+H]^+$ = 329.2 (exact mass = 327.08); $^1H$ NMR (600 MHz, $CDCl_3$): δ 9.28 (s, 1H, –CH=), 8.21 (dd, 1H), 8.16 (dd, 1H), 7.66 (d, 1H), 7.55 – 7.49 (m, 1H), 7.39 (dd, 1H), 7.10 – 7.02 (m, 1H), 6.98 (dd, 1H), 3.88 (s, 3H, $OCH_3$), 2.62 (s, 3H, $CH_3$); $^{13}C$ NMR (151 MHz, $CDCl_3$): δ 164.02, 159.75, 158.11, 155.50, 147.58, 140.39, 134.35, 128.81, 127.37, 126.97, 126.53, 121.03, 121.00, 120.14, 111.44, 55.75, 22.91; Elemental analysis: Calculated for $C_{17}H_{14}ClN_3O_2$ (MW 327.77) 62.30 % C; 4.31 % H; 12.82 % N. Found: 62.52 % C; 4.46 % H; 12.53 % N.

**(*E*)-7-Chloro-3-((3-methoxybenzylidene)amino)-2-methylquinazolin-4(3*H*)-one (39).** White solid; Yield 74 %; m.p. 148.2–148.6 °C; LRMS (APCI) m/z: $[M-H]^-$ = 325.9, $[M+H]^+$ = 328.1 (exact mass = 327.08); $^1H$ NMR (600 MHz, $CDCl_3$): δ 8.97 (s, 1H, –CH=), 8.19 (d, $J$ = 8.5 Hz, 1H), 7.65 (d, $J$ = 2.0 Hz, 1H), 7.49 – 7.45 (m, 1H), 7.43 – 7.38 (m, 3H), 7.14 – 7.07 (m, 1H), 3.88 (s, 3H, $OCH_3$), 2.65 (s, 3H, $CH_3$); $^{13}C$ NMR (151 MHz, $CDCl_3$): δ 166.71, 160.10, 158.25, 155.50, 147.49, 140.59, 134.00, 130.10, 128.77, 127.14, 126.64, 122.35, 120.07, 119.19, 112.55, 55.52, 22.98; Elemental analysis: Calculated for $C_{17}H_{14}ClN_3O_2$ (MW 327.77) 62.30 % C; 4.31 % H; 12.82 % N. Found: 62.72 % C; 4.41 % H; 12.63 % N.

**(*E*)-7-Chloro-3-((4-methoxybenzylidene)amino)-2-methylquinazolin-4(3*H*)-one (40).** White solid; Yield 52 %; m.p. 224.6–225.3 °C; LRMS (APCI) m/z: $[M-H]^-$ = 326.0, $[M+H]^+$ = 328.5 (exact mass = 327.08); $^1H$ NMR (600 MHz, $CDCl_3$): δ 8.85 (s, 1H, –CH=), 8.20 (d, 1H), 7.84 (d, 2H), 7.66 (d, 1H), 7.39 (dd, 1H), 7.00 (d, 2H), 3.89 (s, 3H, $OCH_3$), 2.63 (s, 3H, $CH_3$); $^{13}C$ NMR (151 MHz, $CDCl_3$): δ 166.57, 163.47, 158.28, 155.54, 147.55, 140.43, 130.84, 128.68, 126.97, 126.55, 125.35,

120.14, 114.59, 55.54, 22.77; Elemental analysis: Calculated for $C_{17}H_{14}ClN_3O_2$ (MW 327.77) 62.30 % C; 4.31 % H; 12.82 % N. Found: 62.85 % C; 4.42 % H; 12.52 % N.

**7-Chloro-2-methyl-3-(((1*E*,2*E*)-3-phenylallylidene)amino)quinazolin-4(3*H*)-one (41).** White solid; Yield 86 %; m.p. 198.9–199.7 °C; LRMS (APCI) m/z: $[M-H]^-$ = 321.7, $[M+H]^+$ = 324.2 (exact mass = 323.08); $^1$H NMR (600 MHz, $CDCl_3$): δ 8.69 (d, 1H, –CH=), 8.17 (d, 1H, –CH=), 7.63 (t, 1H, –CH=), 7.57 – 7.52 (m, 2H), 7.44 – 7.35 (m, 4H), 7.20 (d, 1H, CH), 7.09 (dd, 1H, CH), 2.59 (s, 3H, $CH_3$); $^{13}$C NMR (151 MHz, $CDCl_3$): δ 168.93, 158.20, 155.28, 147.52, 146.74, 140.52, 135.10, 130.37, 129.10, 128.75, 127.93, 127.07, 126.60, 123.88, 120.04, 22.92; Calculated for $C_{18}H_{14}ClN_3O$ (MW 323.79) 66.77 % C; 4.36 % H; 12.98 % N. Found: 66.36 % C; 4.49 % H; 12.65 % N.

**(*E*)-7-Chloro-3-((2-fluorobenzylidene)amino)-2-methylquinazolin-4(3*H*)-one (42).** White solid; Yield 73 %; m.p. 197.1–197.8 °C; LRMS (APCI) m/z: $[M-H]^-$ = 313.7, $[M+H]^+$ = 316.4 (exact mass = 315.06); $^1$H NMR (600 MHz, $CDCl_3$): δ 9.32 (s, 1H, –CH=), 8.20 (dd, 1H), 8.17 – 8.11 (m, 1H), 7.65 (d, 1H), 7.58 – 7.51 (m, 1H), 7.40 (dd, 1H), 7.30 – 7.26 (m, 1H), 7.21 – 7.14 (m, 1H), 2.65 (s, 3H, $CH_3$); $^{13}$C NMR (151 MHz, $CDCl_3$) δ 163.56, 161.86, 160.31 (d, $J$ = 5.2 Hz), 158.19, 155.46, 147.45, 140.67, 134.46 (d, $J$ = 8.7 Hz), 128.86, 127.63, 127.19, 126.66, 124.76 (d, $J$ = 3.5 Hz), 120.07, 116.38 (d, $J$ = 20.9 Hz), 23.00.

**(*E*)-7-Chloro-3-((3-fluorobenzylidene)amino)-2-methylquinazolin-4(3*H*)-one (43).** White solid; Yield 42 %; m.p. 194.7–195.5 °C; LRMS (APCI) m/z: $[M-H]^-$ = 313.5, $[M+H]^+$ = 316.2 (exact mass = 315.06); $^1$H NMR (600 MHz, $CDCl_3$): δ 9.11 (s, 1H, –CH=), 8.19 (d, 1H), 7.66 (d, 2H), 7.61 – 7.58 (m, 1H), 7.51 – 7.44 (m, 1H), 7.40 (dd, 1H), 7.28 – 7.22 (m, 1H), 2.66 (s, 3H, $CH_3$); $^{13}$C NMR (151 MHz, $CDCl_3$) δ 164.51 (d, $J$ = 2.9 Hz), 163.93, 162.28, 158.38, 155.57, 147.37, 140.76, 135.09 (d, $J$ = 7.5 Hz), 130.71 (d, $J$ = 8.2 Hz), 128.79, 127.25, 126.69, 125.45 (d, $J$ = 3.1 Hz), 120.04, 119.63 (d, $J$ = 21.6 Hz), 114.56 (d, $J$ = 22.6 Hz), 23.03.

**(*E*)-7-Chloro-3-((4-fluorobenzylidene)amino)-2-methylquinazolin-4(3*H*)-one (44).** White solid; Yield 31 %; m.p. 213.5–214.2 °C; LRMS (APCI) m/z: $[M-H]^-$ = 313.8, $[M+H]^+$ = 316.4 (exact mass = 315.06); $^1$H NMR (600 MHz, $CDCl_3$): δ 8.99 (s, 1H, –CH=), 8.19 (d, 1H), 7.93 – 7.87 (m, 2H), 7.66 (d, 1H), 7.41 (dd, 1H), 7.23 – 7.16 (m, 2H), 2.64 (s, 3H, $CH_3$); $^{13}$C NMR (151 MHz, $CDCl_3$) δ 166.11, 164.97, 164.43, 158.03, 155.16, 147.19, 140.37, 130.83 (d, $J$ = 9.4 Hz), 128.72 (d, $J$ = 3.0 Hz), 128.48, 126.91, 126.38, 119.75, 116.14 (d, $J$ = 22.0 Hz), 22.70 (d, $J$ = 3.4 Hz).

**(*E*)-7-Chloro-3-((4-(dimethylamino)benzylidene)amino)-2-methylquinazolin-4(3*H*)-one (45).** Beige solid; Yield 17 %; m.p. 206.2–206.9 °C; LRMS (APCI) m/z: $[M-H]^-$ = 338.9, $[M+H]^+$ = 341.6 (exact mass = 340.11); $^1$H NMR (600 MHz, $CDCl_3$): δ 8.63 (s, 1H, –CH=), 8.20 (d, 1H), 7.78 – 7.72 (m, 2H), 7.65 (d, 1H), 7.37 (dd, 1H), 6.76 – 6.70 (m, 2H), 3.07 (s, 6H, $N(CH_3)_2$), 2.61 (s, 3H, $CH_3$); $^{13}$C NMR (151 MHz, $CDCl_3$): δ 167.71, 158.37, 155.67, 153.51, 147.67, 140.13, 130.82, 128.62, 126.74, 126.45, 120.20, 119.91, 111.66, 40.07, 22.75; Calculated for $C_{18}H_{17}ClN_4O$ (MW 340.81) 63.44 % C; 5.03 % H; 16.44 % N. Found: 63.18 % C; 4.78 % H; 16.21 % N.

**(*E*)-7-Chloro-3-((2-methylbenzylidene)amino)-2-methylquinazolin-4(3*H*)-one (46).** White solid; Yield 24 %; m.p. 196.8–197.4 °C; LRMS (APCI) m/z: $[M-H]^-$ = 310.5, $[M+H]^+$ = 312.6 (exact mass = 311.08); $^1$H NMR (600 MHz, $CDCl_3$): δ 8.93 (s, 1H, –CH=), 8.19 (d, 1H), 7.73 – 7.70 (m, 1H), 7.69 – 7.63 (m, 2H), 7.42 – 7.34 (m, 3H), 2.64 (s, 3H, $CH_3$), 2.43 (s, 3H, $CH_3$); $^{13}$C NMR (151 MHz, $CDCl_3$): δ 167.27, 158.21, 155.51, 147.52, 140.53, 138.91, 133.59, 132.60, 129.33, 128.97, 128.77, 127.09, 126.61, 126.44, 120.08, 22.97, 21.40; Elemental analysis: Calculated for $C_{17}H_{14}ClN_3O$ (MW 311.77) 65.49 % C; 4.53 % H; 13.48 % N. Found: 65.12 % C; 4.47 % H; 12.93 % N.

**(*E*)-7-Chloro-3-((3-methylbenzylidene)amino)-2-methylquinazolin-4(3*H*)-one (47).** White solid; Yield 36 %; m.p. 152.7–153.5 °C; LRMS (APCI) m/z: $[M-H]^-$ = 310.5, $[M+H]^+$ = 312.7 (exact mass = 311.08); $^1H$ NMR (600 MHz, $CDCl_3$): δ 8.93 (s, 1H, –CH=), 8.20 (d, 1H), 7.74 – 7.70 (m, 1H), 7.69 – 7.63 (m, 2H), 7.42 – 7.34 (m, 3H), 2.64 (s, 3H, $CH_3$), 2.43 (d, 3H, $CH_3$); $^{13}C$ NMR (151 MHz, $CDCl_3$): δ 167.27, 158.22, 155.51, 147.53, 140.54, 138.91, 133.60, 132.60, 129.33, 128.97, 128.77, 127.09, 126.62, 126.44, 120.09, 22.98, 21.40; Elemental analysis: Calculated for $C_{17}H_{14}ClN_3O$ (MW 311.77) 65.49 % C; 4.53 % H; 13.48 % N. Found: 65.31 % C; 4.49 % H; 13.14 % N.

**(*E*)-7-Chloro-3-((4-methylbenzylidene)amino)-2-methylquinazolin-4(3*H*)-one (48).** White solid; Yield 38 %; m.p. 203.9–204.8 °C; LRMS (APCI) m/z: $[M-H]^-$ = 310.1, $[M+H]^+$ = 312.6 (exact mass = 311.08); $^1H$ NMR (600 MHz, $CDCl_3$): δ 8.89 (s, 1H, –CH=), 8.18 (d, 1H), 7.79 – 7.74 (m, 2H), 7.64 (d, 1H), 7.40 – 7.37 (m, 1H), 7.29 (d, 2H), 2.62 (s, 3H, $CH_3$), 2.43 (s, 3H, $CH_3$); $^{13}C$ NMR (151 MHz, $CDCl_3$): δ 167.04, 158.25, 155.52, 147.53, 143.54, 140.48, 129.95, 129.81, 129.01, 128.73, 127.05, 126.59, 120.08, 22.95, 21.85; Elemental analysis: Calculated for $C_{17}H_{14}ClN_3O$ (MW 311.77) 65.49 % C; 4.53 % H; 13.48 % N. Found: 65.27 % C; 4.46 % H; 13.11 % N.

**(*E*)-7-Chloro-3-((2-chlorobenzylidene)amino)-2-methylquinazolin-4(3*H*)-one (49).** White solid; Yield 82 %; m.p. 208.6–209.4 °C; LRMS (APCI) m/z: $[M-H]^-$ = 329.8 $[M+H]^+$ = 332.9 (exact mass = 331.03); $^1H$ NMR (600 MHz, $CDCl_3$): δ 9.53 (s, 1H, –CH=), 8.24 – 8.18 (m, 2H), 7.66 (d, 1H), 7.51 – 7.44 (m, 2H), 7.43 – 7.36 (m, 2H), 2.66 (s, 3H, $CH_3$); $^{13}C$ NMR (151 MHz, $CDCl_3$): δ 163.19, 158.24, 155.49, 147.43, 140.70, 136.56, 133.43, 130.55, 130.35, 128.91, 128.13, 127.32, 127.22, 126.67, 120.10, 23.08; Elemental analysis: Calculated for $C_{16}H_{11}Cl_2N_3O$ (MW 332.18): 57.85 % C; 3.34 % H; 12.65 % N. Found: 57.20% C; 3.25% H; 12.41% N.

**(*E*)-7-Chloro-3-((3-chlorobenzylidene)amino)-2-methylquinazolin-4(3*H*)-one (50).** White solid; Yield 62 %; m.p. 174.7–175.2 °C; LRMS (APCI) m/z: $[M-H]^-$ = 329.9, $[M+H]^+$ = 332.7 (exact mass = 331.03); $^1H$ NMR (600 MHz, $CDCl_3$): δ 9.06 (s, 1H, –CH=), 8.17 (d, 1H), 7.90 (t, 1H), 7.73 – 7.66 (m, 1H), 7.64 (d, 1H), 7.54 – 7.48 (m, 1H), 7.46 – 7.36 (m, 2H), 2.65 (s, 3H, $CH_3$); $^{13}C$ NMR (151 MHz, $CDCl_3$): δ 164.43, 158.35, 155.54, 147.35, 140.77, 135.30, 134.61, 132.51, 130.33, 128.79, 128.17, 127.45, 127.26, 126.66, 120.00, 23.04; Elemental analysis: Calculated for $C_{16}H_{11}Cl_2N_3O$ (MW 332.18) 57.85 % C; 3.34 % H; 12.65 % N. Found: 57.48 % C; 3.31 % H; 12.71 % N.

**(*E*)-7-Chloro-3-((4-chlorobenzylidene)amino)-2-methylquinazolin-4(3*H*)-one (51).** White solid; Yield 54 %; m.p. 246.6–247.2 °C; LRMS (APCI) m/z: $[M-H]^-$ = 329.8, $[M+H]^+$ = 332.7 (exact mass = 331.03); $^1H$ NMR (600 MHz, $CDCl_3$): δ 9.07 (s, 1H, –CH=), 8.19 (d, 1H), 7.84 – 7.79 (m, 2H), 7.66 (d, 1H), 7.50 – 7.44 (m, 2H), 7.40 (dd, 1H), 2.65 (s, 3H, $CH_3$); $^{13}C$ NMR (151 MHz, $CDCl_3$): δ 164.73, 158.37, 155.50, 147.43, 140.73, 138.88, 131.38, 130.01, 129.44, 128.73, 127.19, 126.67, 120.08, 22.86; Elemental analysis: Calculated for $C_{16}H_{11}Cl_2N_3O$ (MW 332.18) 57.85 % C; 3.34 % H; 12.65 % N. Found: 57.36 % C; 3.30 % H; 12.27 % N.

**(*E*)-7-Chloro-3-((2-bromobenzylidene)amino)-2-methylquinazolin-4(3*H*)-one (52).** Beige solid; Yield 61 %; m.p. 208.4–209.7 °C; LRMS (APCI) m/z: $[M-H]^-$ = 374.1, $[M+H]^+$ = 377.2 (exact mass = 374.98); $^1H$ NMR (600 MHz, $CDCl_3$): δ 9.51 (s, 1H, –CH=), 8.23 (dd, 1H), 8.19 (dd, 1H), 7.69 – 7.65 (m, 2H), 7.47 – 7.36 (m, 3H), 2.66 (s, 3H, $CH_3$); $^{13}C$ NMR (151 MHz, $CDCl_3$): δ 165.33, 158.28, 155.52, 147.43, 140.72, 133.62, 133.59, 132.13, 128.94, 128.53, 127.91, 127.23, 126.68, 126.44, 120.12, 23.11; Elemental analysis: Calculated for $C_{16}H_{11}BrClN_3O$ (MW 376.64) 51.02 % C; 2.94 % H; 11.16 % N. Found: 50.36 % C; 3.30 % H; 11.27 % N.

**(*E*)-7-Chloro-3-((3-bromobenzylidene)amino)-2-methylquinazolin-4(3H)-one (53).** Light beige solid; Yield 46 %; m.p. 202.4–203.1 °C; LRMS (APCI) m/z: $[M-H]^-$ =374.0, $[M+H]^+$ = 377.3 (exact mass = 374.98); $^1H$ NMR (600 MHz, $CDCl_3$): δ 9.07 (s, 1H, –CH=), 8.19 (d, 1H), 8.07 (t, 1H), 7.79 – 7.74 (m, 1H), 7.70 – 7.64 (m, 2H), 7.43 – 7.35 (m, 2H), 2.67 (s, 3H, $CH_3$); $^{13}C$ NMR: N/A; Elemental analysis: Calculated for $C_{16}H_{11}BrClN_3O$ (MW 376.64) 51.02 % C; 2.94 % H; 11.16 % N. Found: 50.88 % C; 2.91 % H; 11.08 % N.

## 2.2) Results of antimycobacterial activity

Table S5: Direct antimycobacterial activity comparison between matching pairs from series A and series B.

| CODE | R | log*P* | Antimycobacterial Activity MIC in µg/mL | | | | | |
|---|---|---|---|---|---|---|---|---|
| | | | *Mtb* H37Rv | *Mtb* H37Ra | *M. kans.* | *M. avium* | *M. smeg.* | *M. aurum* |
| 1 | 2,4-diOCH$_3$ | 2.69 | >100 | ≥500 | >100 | ≥500 | ≥500 | ≥500 |
| 15 | | 3.25 | >100 | ≥125 | >100 | ≥125 | ≥125 | ≥125 |
| 2 | 3-OCH$_3$ | 2.82 | 100 | 62.5 | 100 | 62.5 | 250 | 125 |
| 16 | | 3.38 | >100 | 62.5 | >100 | ≥500 | ≥125 | ≥125 |
| 3 | 4-OCH$_3$ | 2.82 | >100 | ≥250 | >100 | ≥250 | ≥250 | ≥250 |
| 17 | | 3.38 | >100 | **7.81** | >100 | ≥500 | **31.25** | **15.625** |
| 5 | 2-CH$_3$ | 3.43 | >100 | ≥500 | >100 | ≥500 | ≥500 | ≥500 |
| 20 | | 3.99 | >100 | ≥250 | >100 | ≥250 | ≥250 | ≥250 |
| 6 | 3-Cl | 3.5 | 50 | **31.25** | **25** | **31.25** | **62.5** | **62.5** |
| 21 | | 4.09 | 25 | **15.625** | >100 | ≥250 | ≥250 | ≥250 |
| 7 | 4-CF$_3$ | 3.86 | >100 | 62.5 | >100 | 125 | 250 | 125 |
| 22 | | 4.42 | >100 | **31.25** | **25** | ≥500 | ≥500 | ≥500 |
| 8 | see Figure 6 | 3.94 | >100 | ≥250 | 50 | 62.5 | ≥500 | 62.5 |
| 23 | | 4.5 | >100 | ≥125 | >100 | ≥125 | ≥125 | ≥125 |
| 11 | 3,4-diCl | 4.06 | >100 | ≥250 | >100 | ≥250 | ≥250 | ≥250 |
| 24 | | 4.62 | >100 | **15.625** | **25** | ≥125 | ≥125 | ≥125 |

## 2.3) Results of cytotoxicity evaluation

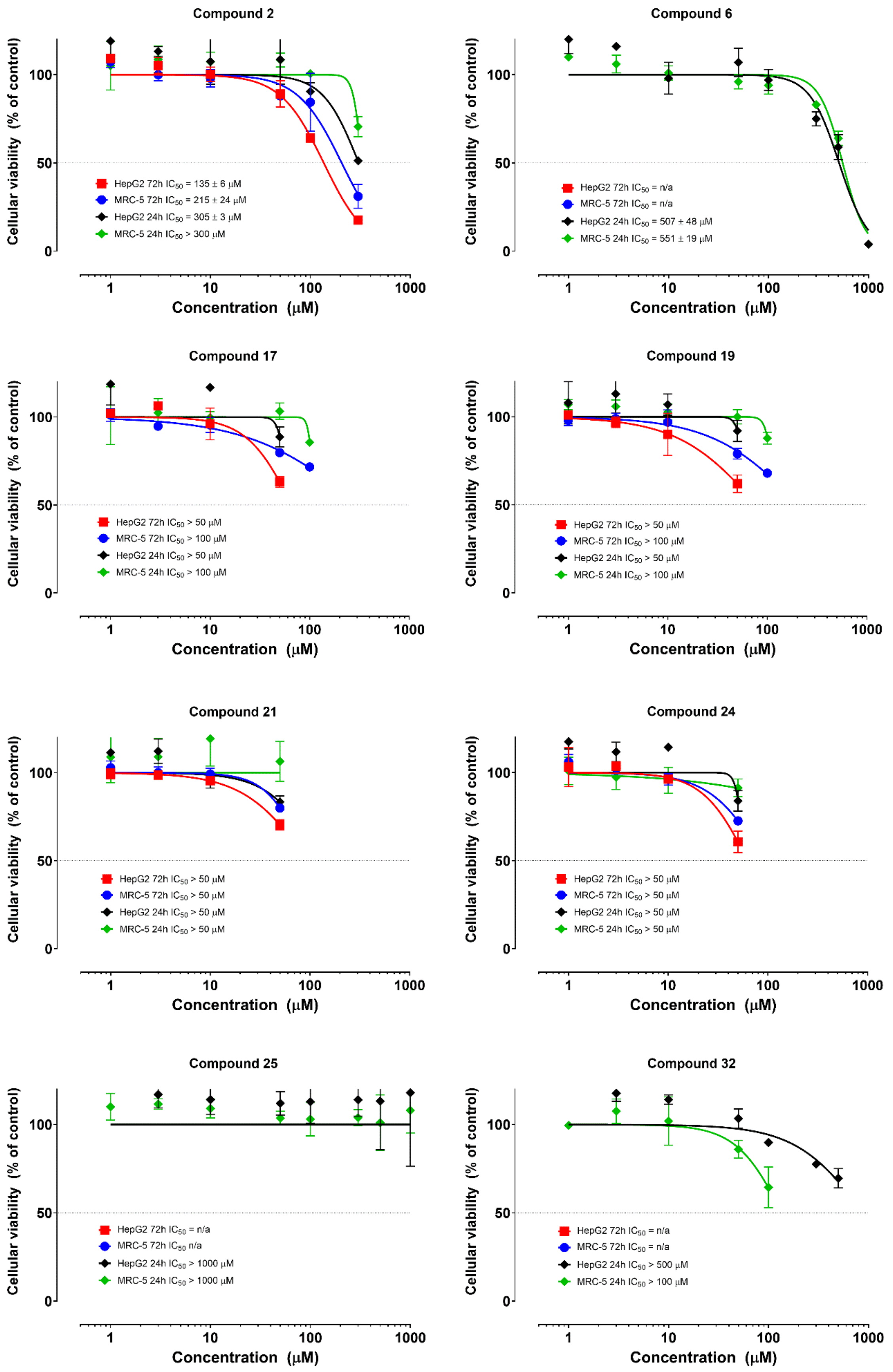


Figure S2.Cellular viability of HepG2 and MRC-5 cel line after treatment with tested compounds

## 2.4) Results of in silico experiments

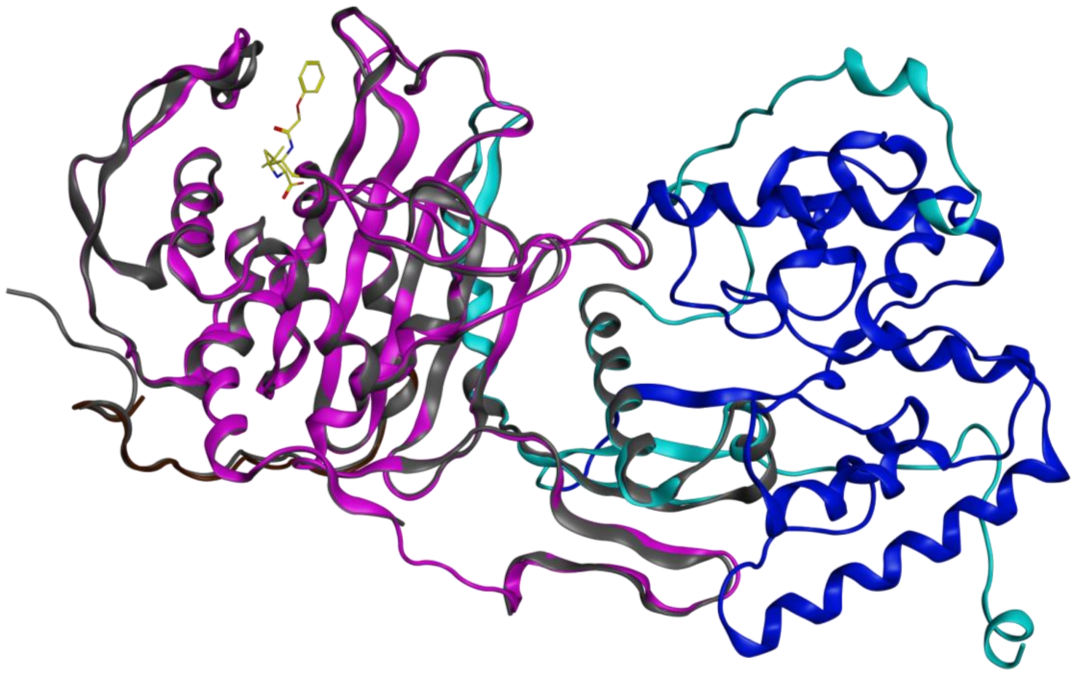

Figure S3. Overlay of AlphaFold model (UniProt ID: P71707, coloured by domains) and crystallographic structure (PDB ID: 5CXW, grey) of mycobacterial Penicillin-binding protein 1A (PonA1). The transpeptidase domain (magenta) can be seen in both structures. The transglycosylase domain (dark blue) and the connecting region (turquoise) can be seen only in the AlphaFold model. Part of the connection between the domains (turquoise) can be seen in the crystallographic structure. The structures were aligned with RMSD 0.446 Å (backbone C, Cα, N).

## 2.4.1) Docking

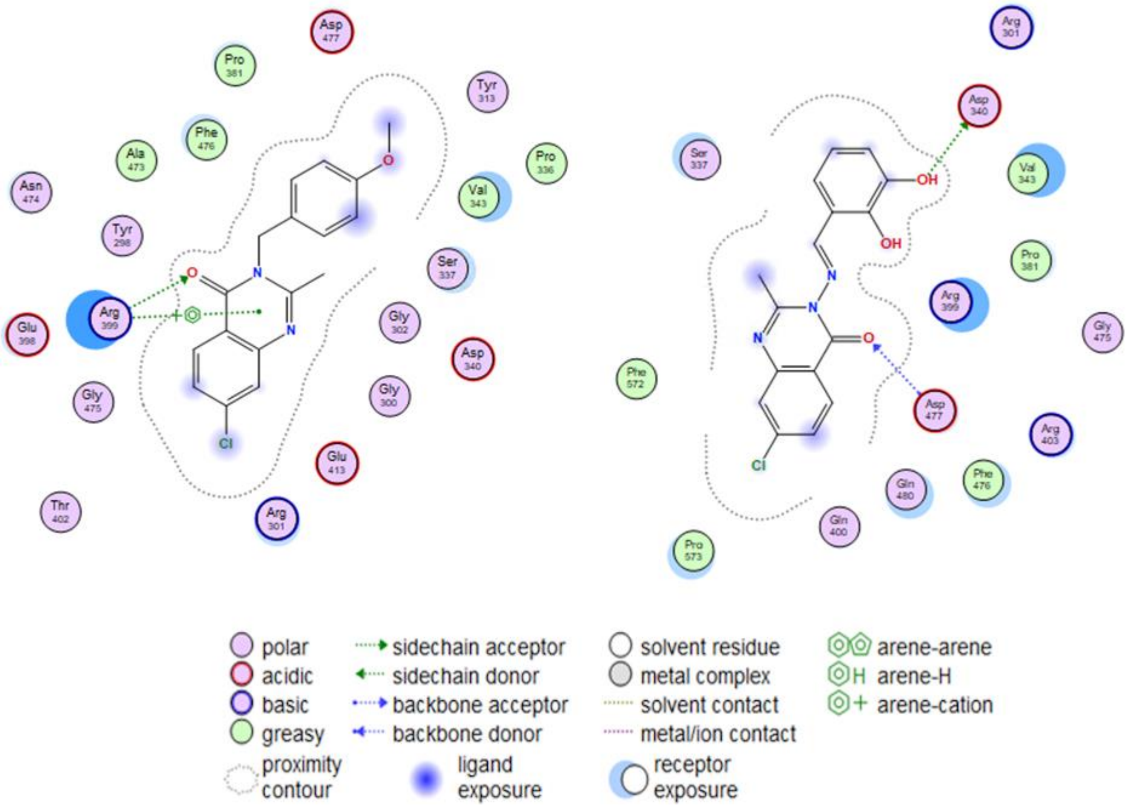


Figure S4. Interaction diagrams of compounds **17** and **32** after docking to AlphaFold model (UniProt ID: P71707)

## 2.4.2) Molecular dynamics – compound 17

Table S6. Occupancy (%) of hydrogen bonds for compound **17**. Only interactions above 5 % of abundance are presented.

| | repl 1 | repl 2 | repl 3 | repl 4 | repl 5 |
|---|---|---|---|---|---|
| ARG399 | 41.29 | 90.18 | 65.67 | 98.19 | 21.94 |
| ARG301 | - | 22.51 | - | 16.75 | - |

Trajectories analyzed by PLIF (Protein-Ligand Interactions Fingerprints) in MOE (Molecular Operating Environment, v2022.02)

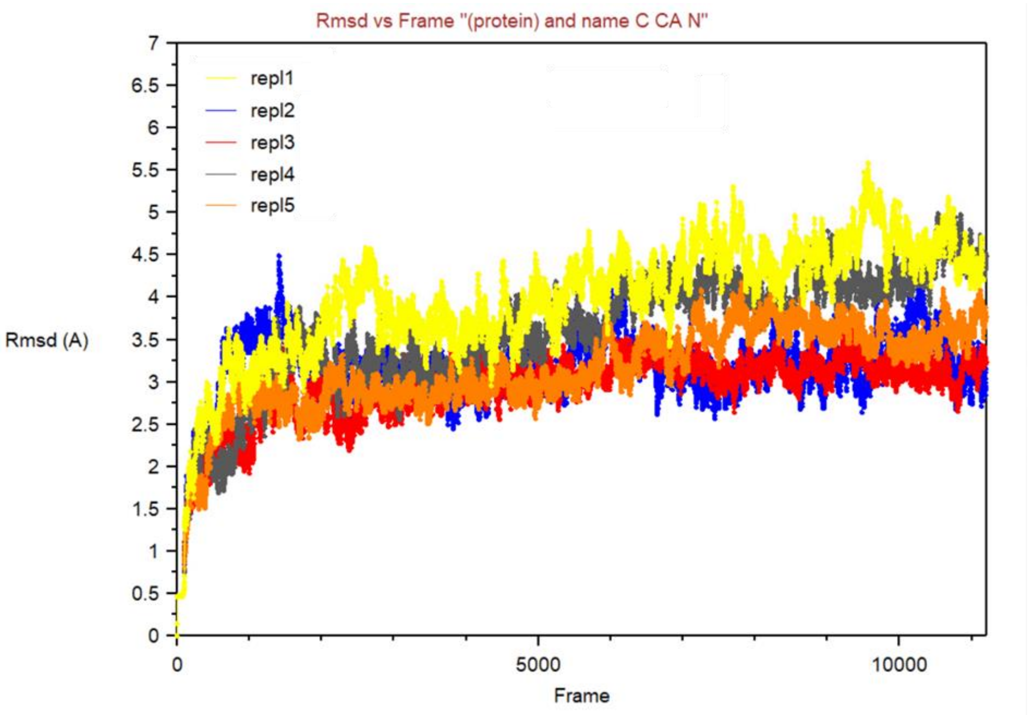


Figure S5. RMSD diagram of the protein backbone (C, Cα, N). The alignment was to the first frame in the system.

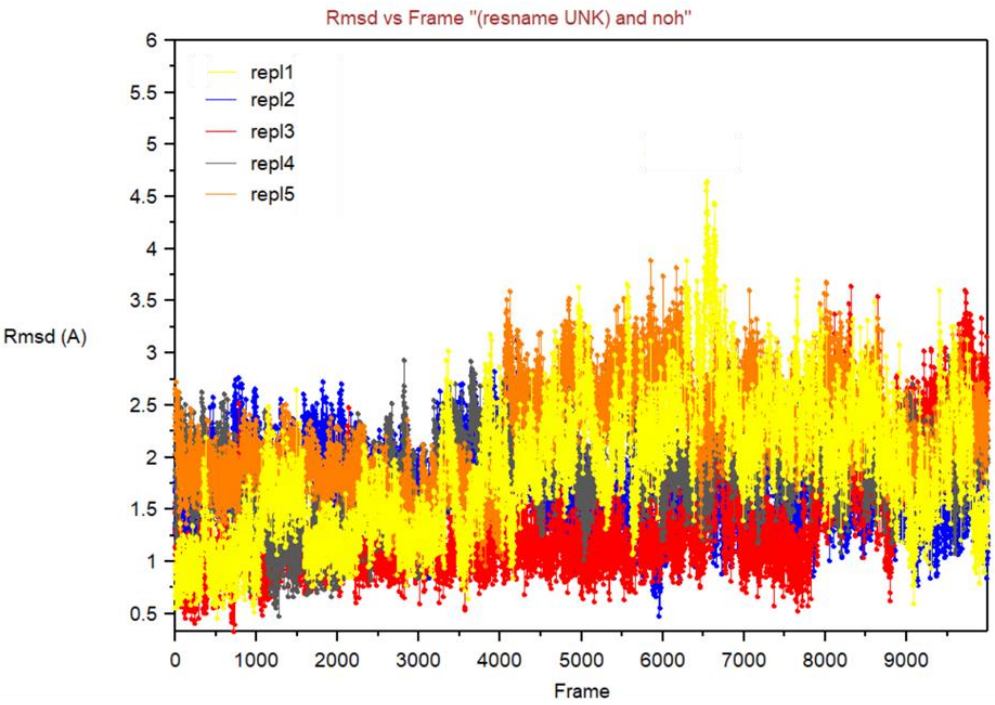


Figure S6. RMSD diagram of compound **17** heavy atoms. The reference is set as the average structure of the production phase.

## 2.4.3) Molecular dynamics – compound 32

Table S7. Occupancy (%) of interactions for compound **32**. Only interactions above 5 % of abundance are presented.

| | Type of interaction | repl 1 | repl 2 | repl 3 | repl 4 | repl 5 |
|---|---|---|---|---|---|---|
| ASP340 | H-bond | 79.87 | 29.95 | 99.76 | 6.42 | 99.84 |
| ASP477 | H-bond | 93.05 | 84.78 | 63.72 | 77.03 | 14.17 |
| ASN474 | H-bond | - | 36.01 | - | 45.64 | - |
| ARG399 | H-bond | 6.07 | 6.88 | 9.18 | 25.09 | 45.64 |
| VAL343 | Arene - H | - | - | 20.91 | - | 5.59 |

Trajectories analyzed by PLIF (Protein-Ligand Interactions Fingerprints) in MOE (Molecular Operating Environment, v2022.02)

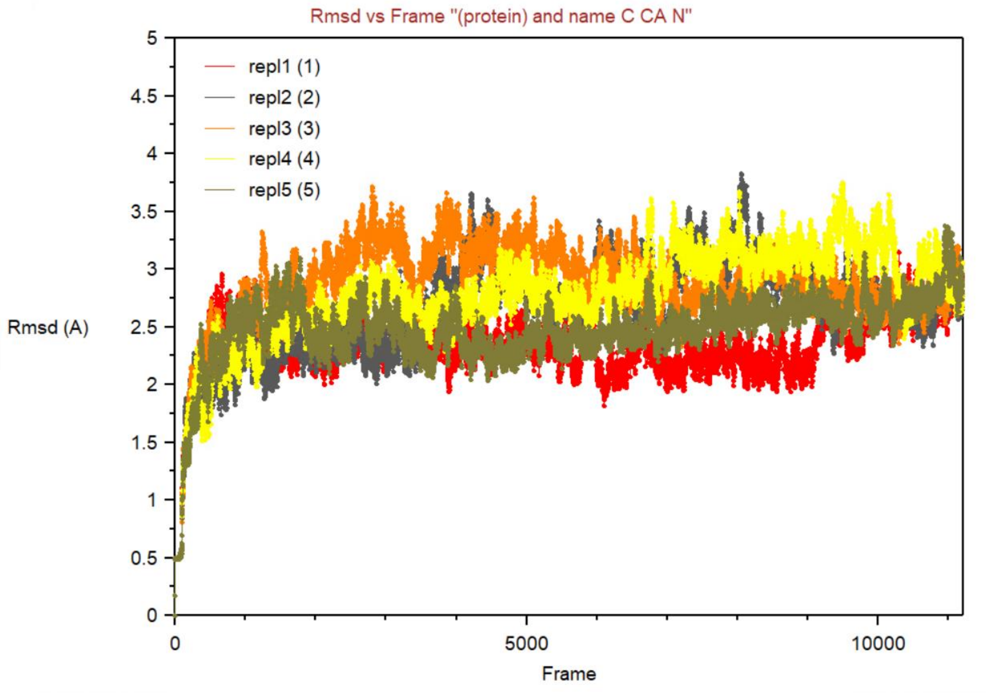


Figure S7. RMSD diagram of the protein backbone (C, Cα, N). The alignment was to the first frame in the system.

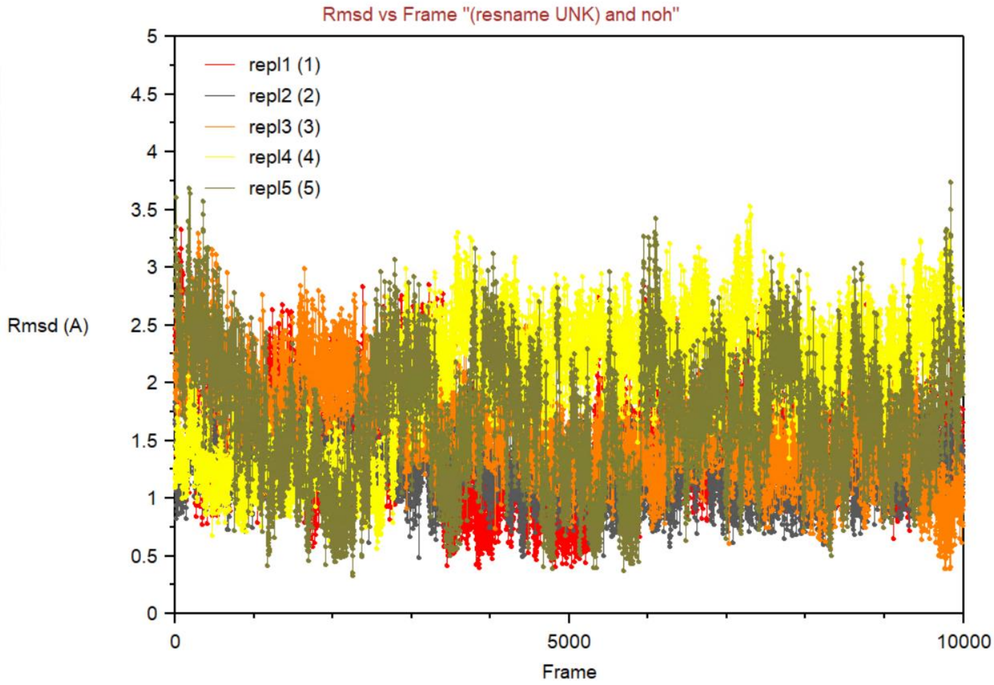


Figure S8. RMSD diagram of compound **32** heavy atoms. The reference is set as average structure of the production phase.

## 2.5) Crystallographic confirmation of double bond configuration

The solid-state structure of compound **47** was studied by single-crystal X-ray diffraction to experimentally determine the configuration of the double bond. Crystals of **47** were grown as colorless needles through slow evaporation of a chloroform solution of the compound. The quinazolinone derivative crystallized in the orthorhombic system, with non-centrosymmetric space group $P2_12_12_1$. Its structure is shown in Figure **S8A**, as thermal ellipsoid diagram. Briefly, the molecule is composed of two flat portions, namely the quinazolinone core and the phenyl group, connected by a double bond in *E* configuration. The geometry of the -N=CH- bond was unambiguously determined by the calculation of the Flack parameter (see Table **S6**), based on the anomalous dispersion effect provided by the chlorine atom. The mean planes of the two rings are inclined at 55.7°; the dihedral angle, measured for C1-N1-N3-C9, is -58.2(6)°. The crystal packing (Figure **S8B**) is mainly ensured by two strong parallel displaced π- π stacking interactions between the quinazolinone and phenyl rings, both separated by a centroid-centroid distance of 4.06 Å. Weak CπH···O and N=CH···O non-traditional H-bonds further contribute to the stabilization of the crystal structure.

**Table S7.** Crystal data and refinement statistics for **47**.

| Identification code | **47** |
|---|---|
| Empirical formula | $C_{17}H_{14}ClN_3O$ |
| Formula weight | 311.76 |
| Temperature (K) | 293(2) |
| Wavelength (Å) | 0.71073 |
| Crystal system | Orthorhombic |
| Space group | $P2_12_12_1$ |
| Unit cell dimensions (Å) | *a* = 4.0581(2) |
| | *b* = 15.4965(11) |
| | *c* = 23.9104(12) |
| Volume (Å$^3$) | 1503.64(15) |
| *Z* | 4 |
| Density calcd. (Mg/m$^3$) | 1.377 |
| Abs. coefficient (mm$^{-1}$) | 0.259 |
| F(000) | 648 |
| Crystal size (mm$^3$) | 0.63 x 0.02 x 0.01 |
| $\vartheta$ range data collection (°) | 1.566 to 24.702 |
| Index ranges | $-3 \le h \le 4$, $-17 \le k \le 18$, $-28 \le l \le 28$ |
| Reflections collected | 11337 |
| Independent reflections | 2561 [$R_{int}$ = 0.0560] |
| Completeness to $\vartheta_{max}$ (%) | 100 |
| Refinement method | Full-matrix least-squares on $F^2$ |
| Data/restraints/parameters | 2561/0/201 |
| Goodness-of-fit on $F^2$ | 1.086 |
| Final R indices [I>2σ(I)] | R1 = 0.0624, wR2 = 0.0922 |
| R indices (all data) | R1 = 0.1016, wR2 = 0.1013 |
| Flack parameter | 0.09(6) |
| Largest diff. peak/hole (e·Å$^{-3}$) | 0.145 and -0.125 |
| CCDC deposition number | 2418965 |

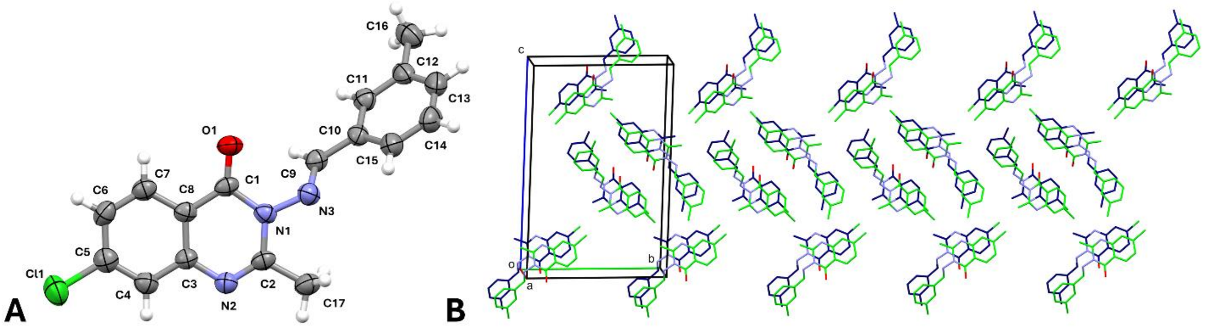


**Figure S9. A.** Thermal ellipsoid diagram of **47**, with the arbitrary atom-numbering scheme. Displacement ellipsoids are drawn at the 40% probability level. **B.** Stick model showing the crystal packing of **47**, viewed along the *a* axis. For the sake of clarity, hydrogen atoms are omitted.

## 2.6) *In silico* prediction of pharmacokinetic properties of selected compounds

Table S8. Pharmacokinetic properties predicted by SwissADME

| Molecule | Compound_17 | Compound_19 | Compound_25 | Compound_32 |
|---|---|---|---|---|
| **MW** | **314.77** | **302.73** | **358.74** | **329.74** |
| #Heavy atoms | 22 | 21 | 25 | 23 |
| #Aromatic heavy atoms | 16 | 16 | 16 | 16 |
| Fraction Csp3 | 0.18 | 0.12 | 0.06 | 0.06 |
| #Rotatable bonds | 3 | 2 | 3 | 2 |
| **#H-bond acceptors** | **3** | **3** | **6** | **5** |
| **#H-bond donors** | **0** | **0** | **1** | **2** |
| MR | 88.22 | 81.69 | 96.26 | 89.46 |
| **TPSA** | **44.12** | **34.89** | **113.3** | **87.71** |
| iLOGP | 3.25 | 3.04 | 2.65 | 2.73 |
| XLOGP3 | 2.97 | 3.1 | 3.16 | 2.42 |
| WLOGP | 3.42 | 3.97 | 2.85 | 2.65 |
| MLOGP | 3.3 | 4.03 | 2.05 | 2.42 |
| Silicos-IT Log P | 4.05 | 4.43 | 1.36 | 3.04 |
| **Consensus Log P** | **3.4** | **3.71** | **2.42** | **2.65** |
| ESOL Log S | -4 | -4.1 | -4.33 | -3.79 |
| ESOL Solubility (mg/ml) | 3.13E-02 | 2.40E-02 | 1.68E-02 | 5.33E-02 |
| ESOL Solubility (mol/l) | 9.93E-05 | 7.91E-05 | 4.67E-05 | 1.62E-04 |
| ESOL Class | Moderately soluble | Moderately soluble | Moderately soluble | Soluble |
| Ali Log S | -3.56 | -3.5 | -5.21 | -3.9 |
| Ali Solubility (mg/ml) | 8.68E-02 | 9.56E-02 | 2.21E-03 | 4.11E-02 |
| Ali Solubility (mol/l) | 2.76E-04 | 3.16E-04 | 6.17E-06 | 1.25E-04 |
| Ali Class | Soluble | Soluble | Moderately soluble | Soluble |
| Silicos-IT LogSw | -6.45 | -6.6 | -4.78 | -4.85 |
| Silicos-IT Solubility (mg/ml) | 1.13E-04 | 7.57E-05 | 5.91E-03 | 4.69E-03 |
| Silicos-IT Solubility (mol/l) | 3.59E-07 | 2.50E-07 | 1.65E-05 | 1.42E-05 |
| Silicos-IT class | Poorly soluble | Poorly soluble | Moderately soluble | Moderately soluble |
| GI absorption | High | High | High | High |
| BBB permeant | Yes | Yes | No | No |
| Pgp substrate | No | No | No | No |
| CYP1A2 inhibitor | Yes | Yes | No | No |
| CYP2C19 inhibitor | Yes | Yes | No | No |
| CYP2C9 inhibitor | Yes | Yes | Yes | No |
| CYP2D6 inhibitor | No | No | No | No |
| CYP3A4 inhibitor | No | No | No | No |
| Lipinski #violations | **0** | **0** | **0** | **0** |
| Ghose #violations | **0** | **0** | **0** | **0** |
| Veber #violations | **0** | **0** | **0** | **0** |
| Egan #violations | **0** | **0** | **0** | **0** |
| Muegge #violations | **0** | **0** | **0** | **0** |
| Bioavailability Score | 0.55 | 0.55 | 0.55 | 0.55 |
| PAINS #alerts | 0 | 0 | 1 | 2 |
| Brenk #alerts | 0 | 0 | 3 | 2 |
| Leadlikeness #violations | 0 | 0 | 1 | 0 |

## 2.7) Results of the similarity searching

The similarity searching against ligands from PDB database did not retrieve any compounds. Similarity searching against the ligands from the ChEMBL database, identified 8 entries with similar ligands (4 unique ligands in total). Two ligands had reported inhibition activity against specific mycobacterial enzymes. Two compounds (1 active) were tested in a whole-cell growth inhibition assay. See table SX for the results.

Table SX. Results of the similarity searching (MACCS [166-bit] structural keys), TI > 0.85} against ChEMBL compounds associated with mycobacterial targets.

| Ligand | Activity | | | | | Target | | |
|---|---|---|---|---|---|---|---|---|
| **ChEMBL ID** | **Standard Type** | **Standard Relation** | **Standard Value** | **Standard Units** | **Comment** | **ChEMBL ID** | **Target Name** | **Target Type** |
| CHEMBL1366795 | $AC_{50}$ | '=' | 60560 | nM | active | CHEMBL1741192 | Replicative DNA helicase | SINGLE PROTEIN |
| CHEMBL1366795 | $EC_{50}$ | '=' | 12000 | nM | active | CHEMBL1741171 | Protein RecA | SINGLE PROTEIN |
| CHEMBL1366795 | $AC_{50}$ | '=' | 15880 | nM | active | CHEMBL1741192 | Replicative DNA helicase | SINGLE PROTEIN |
| CHEMBL1567670 | $EC_{50}$ | '=' | 9920 | nM | active | CHEMBL1741171 | Protein RecA | SINGLE PROTEIN |
| CHEMBL1567670 | $AC_{50}$ | '=' | 13590 | nM | active | CHEMBL1741192 | Replicative DNA helicase | SINGLE PROTEIN |
| CHEMBL1567670 | $AC_{50}$ | '=' | 9285 | nM | active | CHEMBL1741192 | Replicative DNA helicase | SINGLE PROTEIN |
| CHEMBL4442905 | MIC | '>' | 64 | $ug.mL^{-1}$ | | CHEMBL360 | *Mycobacterium tuberculosis* | ORGANISM |
| CHEMBL4444032 | MIC | '=' | 4 | $ug.mL^{-1}$ | | CHEMBL360 | *Mycobacterium tuberculosis* | ORGANISM |

# References

(1) Thomas Schön, Jim Werngren, et al. Antimicrobial susceptibility testing of Mycobacterium tuberculosis complex isolates – the EUCAST broth microdilution reference method for MIC determination, Clinical Microbiology and Infection, Volume 26, Issue 11, 2020, Pages 1488-1492, ISSN 1198-743X, https://doi.org/10.1016/j.cmi.2020.07.036.

(2) Franzblau, S. G.; Witzig, R. S.; McLaughlin, J. C.; Torres, P.; Madico, G.; Hernandez, A.; Degnan, M. T.; Cook, M. B.; Quenzer, V. K.; Ferguson, R. M.; et al. Rapid, low-technology MIC determination with clinical Mycobacterium tuberculosis isolates by using the microplate Alamar Blue assay. Journal of Clinical Microbiology 1998, 36 (2), 362-366. DOI: 10.1128/jcm.36.2.362-366.1998.

(2)European Committee for Antimicrobial Susceptibility Testing (EUCAST) of the European Society for Clinical Microbiology and Infectious Diseases (ESCMID). EUCAST Discussion Document E. Dis 5.1: determination of minimum inhibitory concentrations (MICs) of antibacterial agents by broth dilution. Clin Microbiol Infec 2003; 9:1-7. http://www.eucast.org/documents/publications_in_journals/ (accessed 11 December, 2019).

(3)CLSI, Susceptibility Testing of Mycobacteria, Nocardia spp., and Other Aerobic Actinomycetes, 3 ed. CLSI Standard M24. Wayne, PA: Clinical and Laboratory Standards Institute; 2018.

(4) AST Discussion Document E.Dis 5.1, Clin. Microbiol. Infect., 2003, 9, 1.

(5) EUCAST DEFINITIVE DOCUMENT EDEF 7.3.1. Method for the determination of broth dilution minimum inhibitory concentrations of antifungal agents for yeasts, http://www.eucast.org/fileadmin/src/media/PDFs/EUCAST_files/AFST/Files/EUCAST_E_Def_7_3_1_Yeast_testing__definitive.pdf, (accessed August 2021).

(6) EUCAST DEFINITIVE DOCUMENT E.DEF 9.3.1. Method for the determination of broth dilution minimum inhibitory concentrations of antifungal agents for conidia forming moulds. http://www.eucast.org/fileadmin/src/media/PDFs/EUCAST_files/AFST/Files/EUCAST_E_Def_9_3_1_Mould_testing__definitive.pdf, (accessed August 2021).

(7) M. C. Burla, R. Caliandro, B. Carrozzini, G. L. Cascarano, C. Cuocci, C. Giacovazzo, M. Mallamo, A. Mazzone, G. Polidori, J. Appl. Crystallogr. 2015, 48, 306.

(8) G. M. Sheldrick, Acta Crystallogr., Sect. C: Struct, Chem, 2015, 71, 3.

(9) L. J. Farrugia, J. Appl. Crystallogr. 2012, 45, 849.

(10) M. Nardelli, J. Appl. Crystallogr. 1995, 28, 659.

(11) C. F. Macrae, I. Sovago, S. J. Cottrell, P. T. A. Galek, P. McCabe, E. Pidcock, M. Platings, G. P. Shields, J. S. Stevens, M. Towler, P. A. Wood, J. Appl. Crystallogr. 2020, 53, 226.
